# New Blatter-type radicals from a bench-stable carbene

**DOI:** 10.1038/ncomms15088

**Published:** 2017-05-15

**Authors:** Jacob A. Grant, Zhou Lu, David E. Tucker, Bryony M. Hockin, Dmitry S. Yufit, Mark A. Fox, Ritu Kataky, Victor Chechik, AnnMarie C. O'Donoghue

**Affiliations:** 1 Department of Chemistry, Durham University, South Road, Durham DH1 3LE, UK; 2 Department of Chemistry, University of York, Heslington, York YO10 5DD, UK

## Abstract

Stable benzotriazinyl radicals (Blatter's radicals) recently attracted considerable interest as building blocks for functional materials. The existing strategies to derivatize Blatter's radicals are limited, however, and synthetic routes are complex. Here, we report that an inexpensive, commercially available, analytical reagent Nitron undergoes a previously unrecognized transformation in wet acetonitrile in the presence of air to yield a new Blatter-type radical with an amide group replacing a phenyl at the C(3)-position. This one-pot reaction of Nitron provides access to a range of previously inaccessible triazinyl radicals with excellent benchtop stabilities. Mechanistic investigation suggests that the reaction starts with a hydrolytic cleavage of the triazole ring followed by oxidative cyclization. Several derivatives of Nitron were prepared and converted into Blatter-type radicals to test the synthetic value of the new reaction. These results significantly expand the scope of using functionalized benzotriazinyls as stable radical building blocks.

## Abstract (web summary)

Blatter's radicals are highly valuable stable organic free radicals, comprising a heterocyclic ring system functionalized with a heteroaryl or alkyl group at the C-3 position. Here, the authors report a synthetic strategy to obtain Blatter's radical substituted with an amine and amide group.

Blatter's radical **1** (Fig. 1)^1^ is an example of a benzotriazinyl radical with remarkable bench-top stability. Although relatively unexplored since the early report by Blatter in 1968, there has been increased recent interest in this radical and derivatives due to their suitability as building blocks for magnetic materials^2^,^3^,^4^,^5^,^6^,^7^,^8^,^9^,^10^,^11^,^12^,^13^, as polymerization initiators^14^,^15^,^16^ and as ligands in novel radical-metal coordination complexes^17^,^18^. Related benzotriazines and N-oxide derivatives have seen application in medicinal chemistry, including as anti-cancer drugs, where benzotriazinyl radicals have been proposed as potential DNA-damaging species^19^,^20^,^21^,^22^. All reported Blatter-type radicals to date limit C(3)-substitution to (hetero)aryl, alkenyl or alkyl groups.

Here, we report the mechanism and initial scope of an unexpected reaction of Nitron **2** (a low cost, common analytical reagent) to give a new kind of Blatter-type radical with an amide replacing a phenyl at the 3-position (Fig. 1). Nitron **2** has been used for gravimetric and spectrophotometric determination of nitrate and perchlorate ions for over 100 years^23^, and is usually assigned a zwitterionic Lewis structure **2**. In 2012, Färber *et al*.^24^ provided evidence that Nitron exhibits some reactivity more consistent with N-heterocyclic carbene (NHC) tautomer **2**′, which had not been previously considered. Although the levels of NHC tautomer were too small to be detectable by NMR, Nitron **2** reacted with typical trapping reagents for nucleophilic carbenes including elemental sulfur and CS_2_.

## Results

### Transformation of Nitron 2 to Blatter-type radical 3

During the course of our studies we found that Nitron **2** undergoes an unprecedented one-pot conversion into amido Blatter radical **3** which can subsequently be hydrolysed to yield an amine-functionalized radical **4**, thus providing useful structural handles to enable modification of the properties for functional materials and other applications (Fig. 2). Our initial aims were to investigate the proton transfer properties of Nitron **2** compared with related 1,2,4-triazolium ions to further explore the significance of the **2**–**2′** tautomerization. We have recently studied the proton transfer reactions of a broad range of triazolium salt precursors of triazolyl NHCs structurally very similar to the NHC tautomer of Nitron **2′**, albeit with alkyl or aryl rather than an anilino-substituent^25^,^26^. In a similar study of the proton transfer properties of Nitron, it was noted that the colour of stock solutions of Nitron **2** in acetonitrile changed from translucent orange to opaque brown over 24 h. By contrast, analogous acetonitrile solutions of all other triazolium ions that we have studied to date remained unchanged over several weeks. Black crystals were observed to form upon concentration of the stock acetonitrile solution of Nitron **2**, and after isolation and purification, the unusual decomposition product **3** was isolated in 58% yield. Radical **3** proved to be stable in air and at room temperature for months, and its structure was determined using X-ray crystallography (Fig. 3a).

The structure of amido radical **3** (Fig. 3a) contains two crystallographically independent molecules with different orientation of Ph-substituents at N1. Molecules of **3** stack along the *a*-direction (Supplementary Fig. 1) and this slipped-stacked packing motif has been found in the other known crystal structures of Blatter-type radicals^27^. Two independent molecules alternate in the stack with corresponding interplanar distances between the r.m.s. planes of benzotriazines of 3.68 and 3.61 Å (Supplementary Fig. 2). The shortest C…N and C…C interatomic contacts between planes are equal to 3.62 and 3.53 Å (Fig. 3a), similar to those reported for other benzotriazinyl radicals (3.3–3.8 Å). Additional C–H…O and C–H…π contacts link the stacks together into a 3D-framework. Manipulation of weak hydrogen bonding interactions, as observed in **3** (Fig. 3a), may provide a means for controlling bulk magnetic behaviour^28^,^29^.

EPR studies of **3** revealed hyperfine coupling constants (hfccs) similar to those reported for related Blatter radicals and peak intensities consistent with pure radical character. The solution EPR spectrum of **3** (Fig. 3b) exhibits nine lines consistent with the coupling of the unpaired electron with the three ring triazinyl nitrogens. The largest hfcc can be assigned to N(1) (8.14 G) with the next largest (5.01 G, 4.69 G) to N(2) and N(4), respectively (Supplementary Tables 1–3). The SOMO and spin distributions of **3** determined by computations show the free radical to largely reside at the nitrogens of the triazine ring (N1, N2 and N4) (Fig. 3c,d, Supplementary Fig. 3), and the data are in accord with reported calculated data for related radical species^3^,^6^. The hfcc for the fourth exo-cyclic nitrogen is unresolved. The redox behaviour of radical **3** is typical of 1,2,4-benzotriazinyls showing two fully reversible waves that correspond to −1/0 and 0/+1 processes (Supplementary Fig. 4, Supplementary Table 4).

### Synthesis of amino-substituted Blatter-type radical 4

As well as amide radical **3**, we isolated trace amounts of green amino radical **4** (Fig. 2) upon column purification of the stock solution of Nitron **2** in acetonitrile. Subsequently, it was shown that **4** can be prepared from **3** in 70% yield by hydrolysis in concentrated sodium hydroxide solution. The structure of radical **4**, which lacks a formyl group on the C(3)-nitrogen substituent, was confirmed by X-ray crystallography (Supplementary Fig. 5), EPR (Supplementary Fig. 6), cyclic voltammetry (Supplementary Fig. 7) and other techniques. The SOMO and spin distribution of radical **4** are similar to that of **1** and **3** (Supplementary Fig. 8). Our observation of the clean conversion of radical **3** to **4**, albeit under strongly basic conditions, suggests that the formation of **4** during the reaction of Nitron **2** in acetonitrile is due to hydrolysis of **3**. To our knowledge, this is also the first report of a C(3)-amino substituted Blatter radical **4**, which could be a useful and versatile building block for the preparation of other related radicals.

### Reaction mechanism and optimization of reaction conditions

A potential mechanism for the formation of radical **3** from Nitron **2** is proposed in Fig. 4. This mechanism relies upon initial partial hydrolysis by adventitious water in acetonitrile. Attack of water at the carbenoid centre of Nitron **2** followed by subsequent ring-opening would yield amido-substituted amidrazone (*Z*)-**5**, which can easily isomerize to (*E*)-**5**. In the case of Nitron **2**, the presence of tautomer **2**′ could favour the initial hydration step that precedes ring-opening. Amidrazones such as (*E*)-**5** are also intermediates in the conventional synthetic route to Blatter-type radicals from imidoyl or hydrazonyl chlorides^2^,^3^,^6^,^30^,^31^,^32^. Similar to the literature-proposed mechanism^30^,^31^,^32^, conversion of (*E*)-**5** to **3** could thus involve the initial *in situ* formation of 1,2,4-triazabutadienes **6**, followed by electrocyclic ring closure to benzotriazines and further oxidation. Alternatively, the amidrazone could undergo one-electron oxidation to a hydrazonyl radical **7** followed by ring closure to a benzotriazine and further oxidation (Fig. 4).

The proposed mechanism for the conversion of Nitron **2** into radical **3** was clarified in a range of experiments. No EPR signal could be obtained when a solution of Nitron **2** in dry acetonitrile was exposed to UV light for 4 h or when stirred under an oxygen atmosphere for 3 days. By contrast, an EPR signal could be detected after addition of 2% v/v water to the acetonitrile solution confirming the requirement for low levels of water. Triazabutadiene **8** (Fig. 5) was isolated in 51% yield from the reaction of Nitron **2** in aqueous acetonitrile (50% v/v) with no added base. The yield of **8** increased to 75% upon addition of aqueous KOH (0.5 M). Trace amounts of triazabutadiene **8** (<1%) were also identified in the reaction of Nitron **2** in acetonitrile only. Anilino-substituted triazabutadienes are known oxidation products of corresponding amidrazones^33^,^34^. Early reports of the usage of Nitron in alkaline solution suggest formation of **8** (refs 23, 35). The isolation of **8** thus supports the proposed initial hydrolytic ring-opening of Nitron **2** (Fig. 4).

The structure of **8** obtained by X-ray crystallography (Supplementary Fig. 9) shows N^1^=N^2^ and C^3^=N^4^ in an *s*-*trans* conformation. The corresponding *s*-*cis* conformer **8**′ is directly analogous to **6** and hence a possible precursor to amino radical **4**. Rotational barriers of 3.3, 5.4 and 3.5 kcal mol^−1^ were calculated for triazabutadienes **6**, **8** and **9**, the potential precursors to radicals **3**, **4** and **1** (Supplementary Fig. 10). A recent computational study of the electrocyclization of *s*-*cis* **9** reveals a high 38.6 kcal mol^−1^ barrier, which is substantially greater than the computed rotational energy barriers above^3^. This suggests that the *s*-*trans*/*s*-*cis* C–N rotational barrier is not limiting in the cyclization of these triazabutadienes.

Stirring **8** overnight in acetonitrile did not give any detectable formation of **4** even in the presence of equimolar Ag_2_O and NaOH (1 M). This supports a post-cyclization route for the formation of trace quantities of amino radical **4** from Nitron **2** under these experimental conditions from the subsequent hydrolysis of amido radical product **3**. The stability of triazabutadiene **8**, however, does not exclude the intermediacy of N-formyl triazabutadiene **6** in the formation of **3**. We speculate that N-formylation of **8** could be essential to facilitate electrocyclization, and the mild hydrolysis of Nitron **2** provides selective access to **6**.

An acetonitrile solution of 1,2,4-triphenyltriazolium tetrafluoroborate **10** remained unchanged for at least 10 days at room temperature (Supplementary Fig. 11). Similarly, 99% v/v acetonitrile solutions of **10** containing 1% H_2_O and equimolar Ag_2_O and NaOH (1 M) showed no evidence of radical formation by EPR when left overnight at room temperature. These data highlight the importance of the exocyclic nitrogen on Nitron **2** to enable conversion to Blatter-type radicals. The presence of nitrogen in Nitron **2** presumably favours the initial hydration/ring-opening step and also stabilizes the radical product.

To optimize the conversion of **2** to **3**, we screened a range of experimental conditions. Addition of small amounts of water (≤10 v/v%) to a solution of Nitron **2** in acetonitrile was found to improve the yield of radical **3**; however, higher volumes resulted in significantly lower yields of radical and gave triazabutadiene **8** as major product (see Supplementary Methods). The oxidative cyclization of amidrazones is known to be accelerated by a variety of oxidants and catalysts^30^,^36^,^37^,^38^,^39^,^40^,^41^,^42^. Addition of Ag_2_O or PbO_2_ and base to acetonitrile solutions of **2** increased the rate of conversion although yields were similar. Additional solvents were explored (see Supplementary Methods); however, it appears that acetonitrile is fortuitously best for the conversion of Nitron **2** to radical **3.** Optimal yields were obtained in the presence of 1% v/v water as co-solvent (82% after 72 h).

### Scope of the new transformation

**Figure 1:**
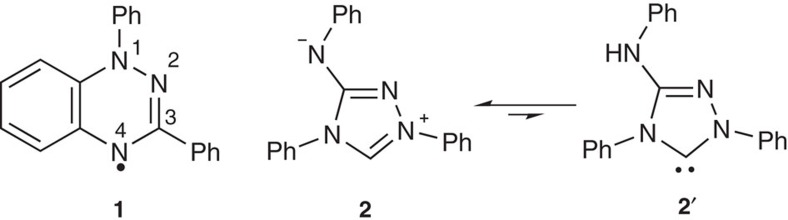
Blatter's radical and Nitron tautomers. Blatter's radical **1** was first isolated in 1968 and subsequent synthetic approaches limited C(3) substitution to aryls/alkyls/alkenyls. Nitron is a commercially available analytical reagent with zwitterionic **2** and carbenic **2′** tautomeric forms.

**Figure 2:**
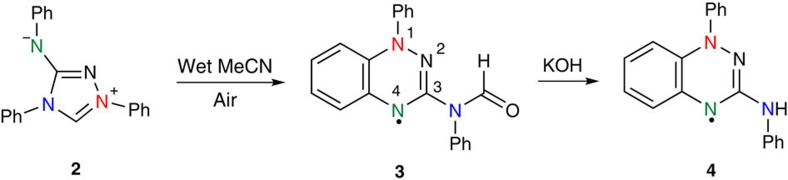
New transformation of analytical reagent Nitron. Conversion of Nitron **2** in wet acetonitrile to C(3)-amido Blatter radical **3**. The benzotriazinyl radical component remains intact in the subsequent hydrolysis of **3** to novel C(3)-amino radical **4** in strongly basic solution.

**Figure 3:**
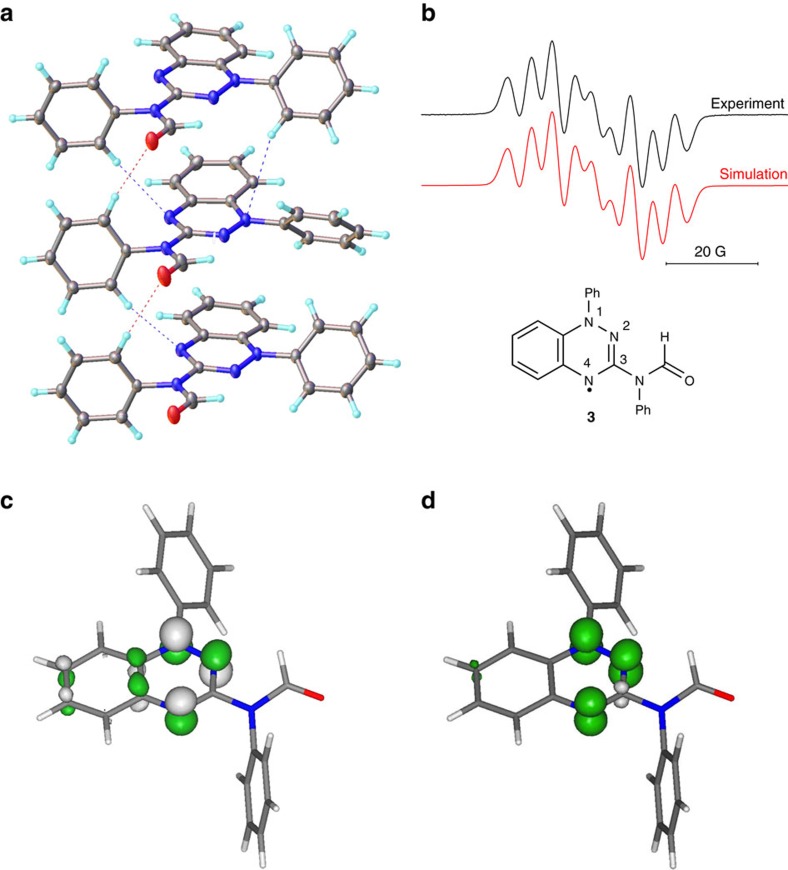
Characterization of C(3)-amido Blatter-type radical. (**a**) X-ray crystal structure of **3** showing two orientations of the phenyl group in the solid state and weak hydrogen bonds in the stack of molecules. (**b**) EPR spectrum of **3** in dioxane at 343 K. (**c**) SOMO in **3** with contours plotted at ±0.07 (e/bohr^3^)^1/2^. (**d**) Spin density distribution in **3** with contours plotted at ±0.008 (e/bohr^3^)^1/2^.

**Figure 4:**
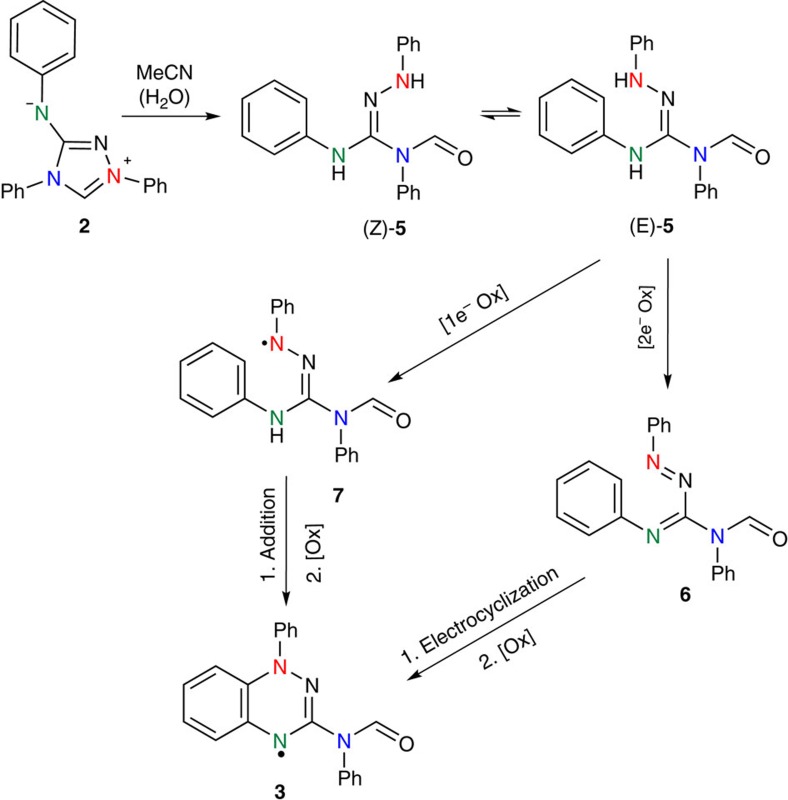
Proposed mechanism for the formation of C(3)-amido Blatter-type radical from Nitron. The first step involves initial hydrolytic ring-opening of Nitron **2** to amidrazones **5**. Two electron oxidation of **5** to C(3)-amidotriazabutadiene **6** and subsequent oxidative electrocyclization would yield C(3)-amido Blatter radical **3**. Alternatively the one electron oxidation of **5** to hydrazonyl radical **7** followed by addition to the adjacent benzene ring and further oxidation would also yield **3**.

**Figure 5:**
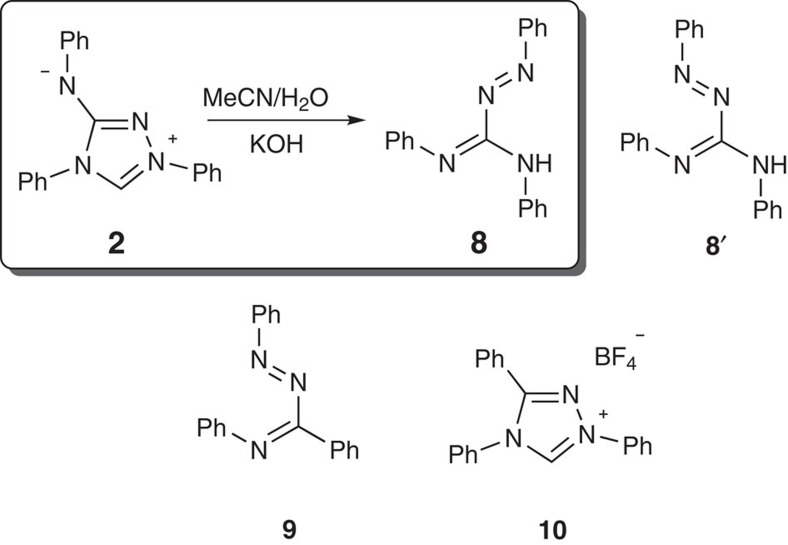
Mechanistic insights. Conversion of Nitron **2** to s-*trans*-3-anilino triazabutadiene **8** in aqueous acetonitrile under alkaline conditions, and the structures of *s*-*cis*-3-anilino triazabutadiene **8′**, *s*-*cis*-3-phenyl triazabutadiene **9** and triphenyltriazolium tetrafluoroborate **10**.

**Figure 6:**
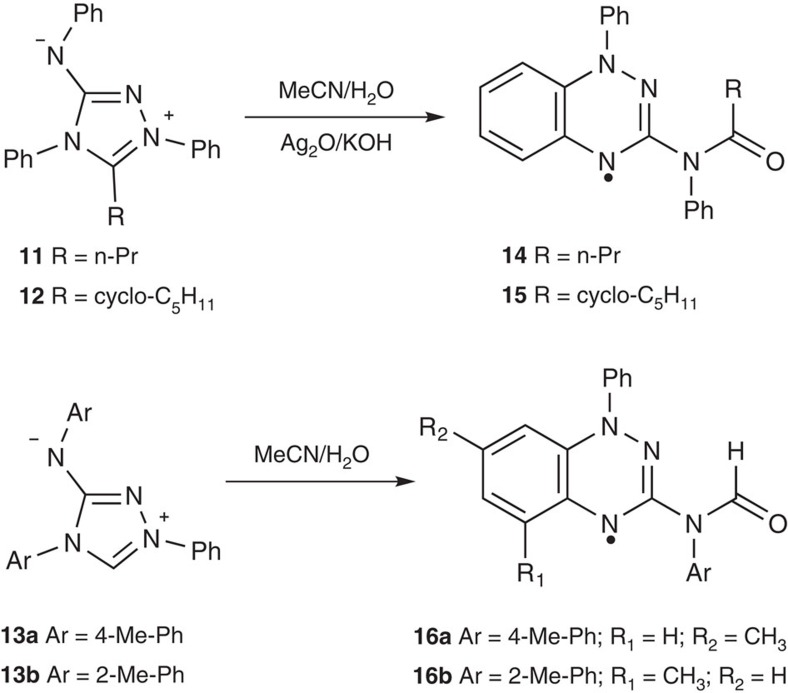
Exploring the scope of the new transformation. Conversion of novel Nitron derivatives **11**–**13** to new Blatter-type radicals **14**–**16**.

Our new facile one-pot preparation of radical **3**, and novel substitution pattern, prompted us to explore other Nitron derivatives as substrates. Using the most recently reported literature synthesis of Nitron **2** (ref. 43), four additional derivatives **11**–**13** (Fig. 6) were prepared (see Supplementary Methods). Importantly, all Nitron derivatives could be converted to corresponding Blatter radicals **14**–**16** in one pot in 42–67% yields using the optimized conditions described above. Conversion of Nitron derivatives **11** and **12** to corresponding amido radicals **14** and **15** in acetonitrile required oxidant and base for significant reaction to occur overnight. By contrast, Nitron derivatives **13a** and **13b** were unstable and converted spontaneously to radicals **16a** and **16b**. We speculate that spontaneous conversion of **13a,b** into Blatter-type radicals is facilitated by easy tautomerization (cf. Fig. 1), which is not possible for **11** or **12**. These initial results highlight the potential of the route to a broader range of stable C(3)-amido-substituted Blatter radicals.

In summary, a novel simple one-pot reaction of Nitron **2** to give new stable radical **3** was observed in acetonitrile, which is the first example to our knowledge of a Blatter-type system with an amido-substituent at C(3). Such derivatives are likely to be challenging to access by conventional routes. Conversion of amido radical **3** to amino radical **4** was achieved in strongly basic solutions in good yield. Remarkably, the benzotriazinyl radical component remained unaltered under these conditions highlighting the intrinsic stability of these radicals. As well as the potential for more diverse properties, this alternative substitution of **3** and **4** opens up new avenues for modification of such radical systems towards functional materials, and for the incorporation of the benzotriazinyl radical system into larger molecular assemblies. Our preliminary synthetic studies of derivatives of Nitron provided access to additional novel radicals **14–16**. We propose that the formation of radical **3** depends on the initial hydrolytic ring opening of Nitron **2** to amidrazone **5** followed by oxidative cyclization. Future work will explore the full scope of this new reaction, and using the novel C(3)-amido and amino functionalities as handles for further modification.

## Methods

### Optimized method for conversion of Nitron **2** to C(3)-amido radical **3**

Nitron (0.99 g, 16 mmol) was dissolved in acetonitrile (99 ml) along with 1% water (1 ml) and stirred with exposure to air for 72 h. The solution was then dried *in vacuo* to give crude black crystals. Column chromatographic purification of the crude black crystals (98:2 DCM:MeOH), where the major product **3** eluted as a dark red fraction, followed by concentration under reduced pressure, yielded a black solid. The solid was then recrystallized from minimum hot ethanol to yield amido radical **3** as black crystals (0.85 g, 82%). m.p. 143–145 °C; Elemental analysis: Calcd. For C_20_H_15_N_4_O: C, 73.38; H, 4.62; N, 17.11; Found: C, 73.19; H, 4.65; N, 17.07; IR: 3,070, 2,925, 1,681, 1,586, 1,483, 1,370, 1,204, 1,077, 841, 760, 692, 612, 552, 495 cm^−1^; MS (ESI, m/z, %): 327 (M^+^, 100%), 328 ([M+H]^+^, 69.53%), 329 ([M+2H]^+^, 39.99%), 330 ([M+3H]^+^, 4.91%). HRMS (ESI): Calcd. For C_20_H_15_N_4_O: 327.1246; Found: 327.1252 (within 1.0 p.p.m.).

### Data availability

Data supporting the findings of this study are available within the article and its Supplementary Information files. For the experimental and computational procedures, and spectroscopic and physical data of compounds, see Supplementary Methods. The CCDC 1411555, CCDC 1484332 and CCDC 1411554 contain the crystallographic data for compounds **3**, **4** and **8**, respectively (Supplementary Figs 1, 2, 5 and 9, Supplementary Table 7). These data can be obtained free of charge from the Cambridge Crystallographic Data Center (www.ccdc.cam.ac.uk). For cyclic voltammograms of **3** and **4**, see Supplementary Figs 4 and 7, and Supplementary Table 4. For EPR spectra of compounds **4**, **14**, **15**, **16a** and **16b**, see Supplementary Figs 6 and 12. For IR and UV-Vis spectra of **3** and **4**, see Supplementary Figs 13–15 and Supplementary Tables 5 and 6. For mass spectra of the compounds in this article, see Supplementary Figs 16–31. For NMR spectra of the compounds in this article, see Supplementary Figs 32–44.

## Additional information

**How to cite this article:** Grant, J. A. *et al*. New Blatter-type radicals from a bench-stable carbene. *Nat. Commun.* **8,** 15088 doi: 10.1038/ncomms15088 (2017).

**Publisher's note**: Springer Nature remains neutral with regard to jurisdictional claims in published maps and institutional affiliations.

## Supplementary Material

Supplementary Information Supplementary figures, supplementary tables, supplementary methods and supplementary references.
