# Supplementary material for: New Blatter-type radicals from a bench-stable carbene
Source: Nat Commun. 2017 May 15;8:15088. doi: 10.1038/ncomms15088 (PMC5440670; doi:10.1038/ncomms15088)
Supplement: Supplementary Information — Supplementary figures, supplementary tables, supplementary methods and supplementary references. [file ncomms15088-s1.pdf]

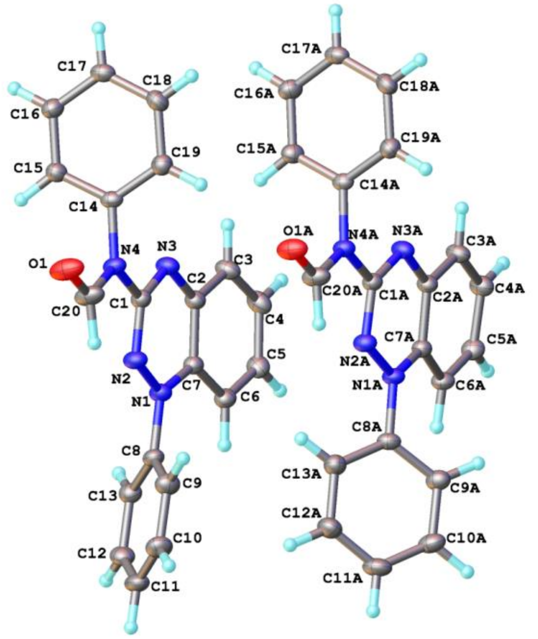

Supplementary Figure 1. Two independent molecules of **3**.

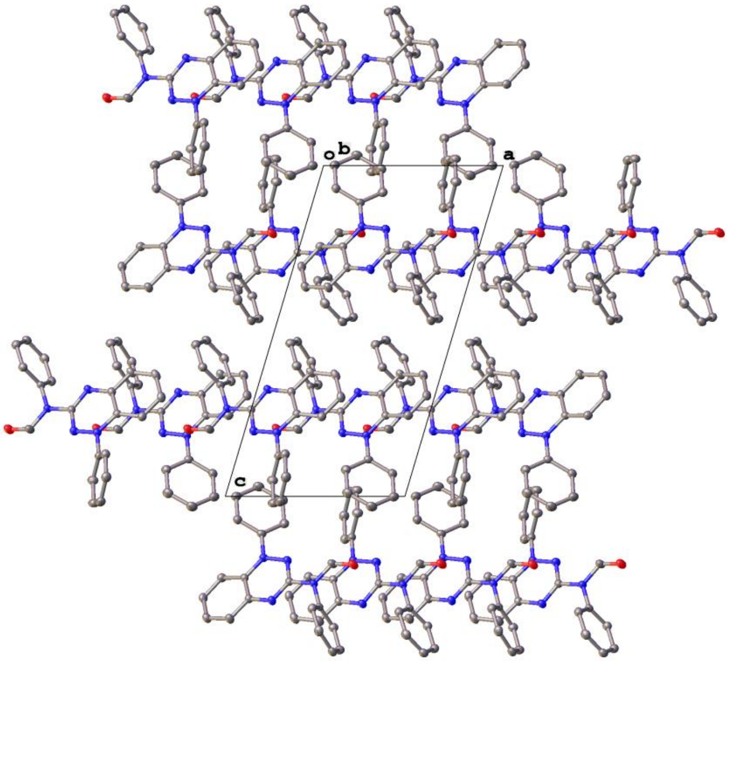

Supplementary Figure 2. Crystal structure of **3** showing molecules stacked along the α-direction in a slipped stack packing motif.

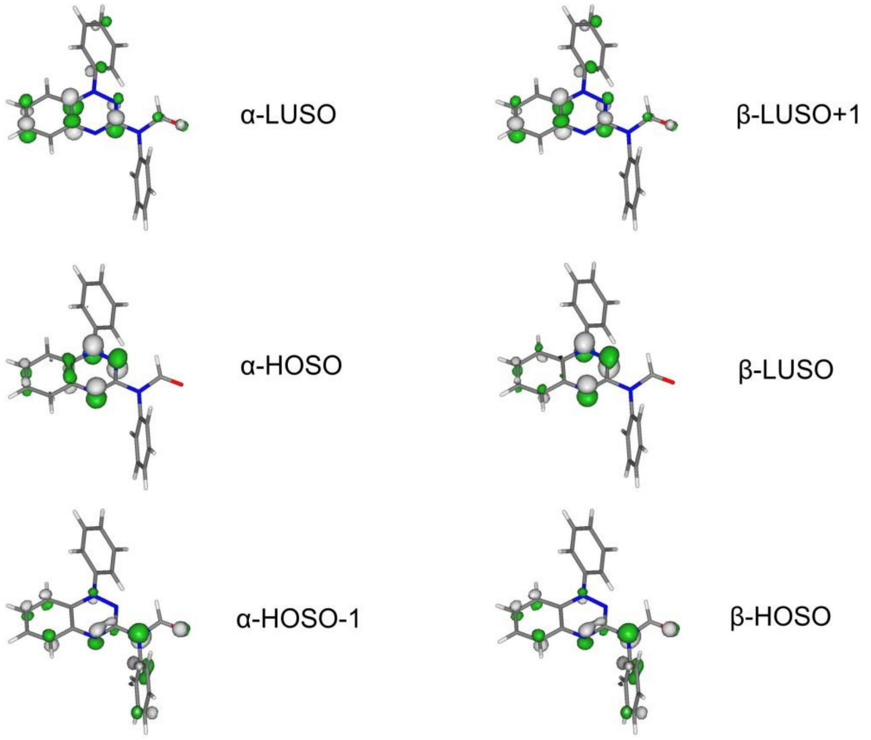

Supplementary Figure 3. Pertinent orbitals in the radical species **3**. SOMO in the radical species is assumed here to be the highest alpha(α) singly occupied molecular orbital, α-HOSO.

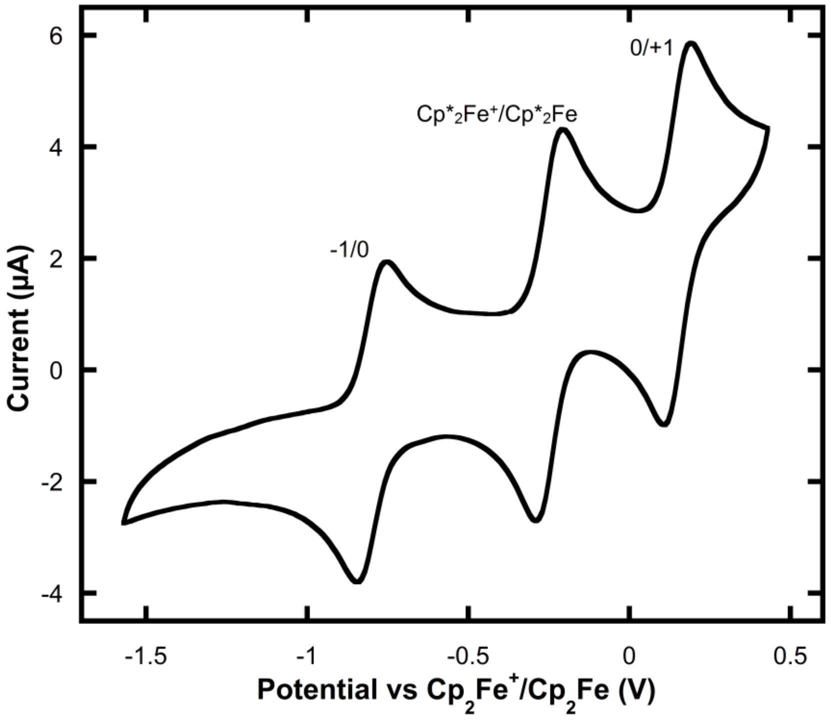

Supplementary Figure 4. Cyclic voltammogram of **3**.

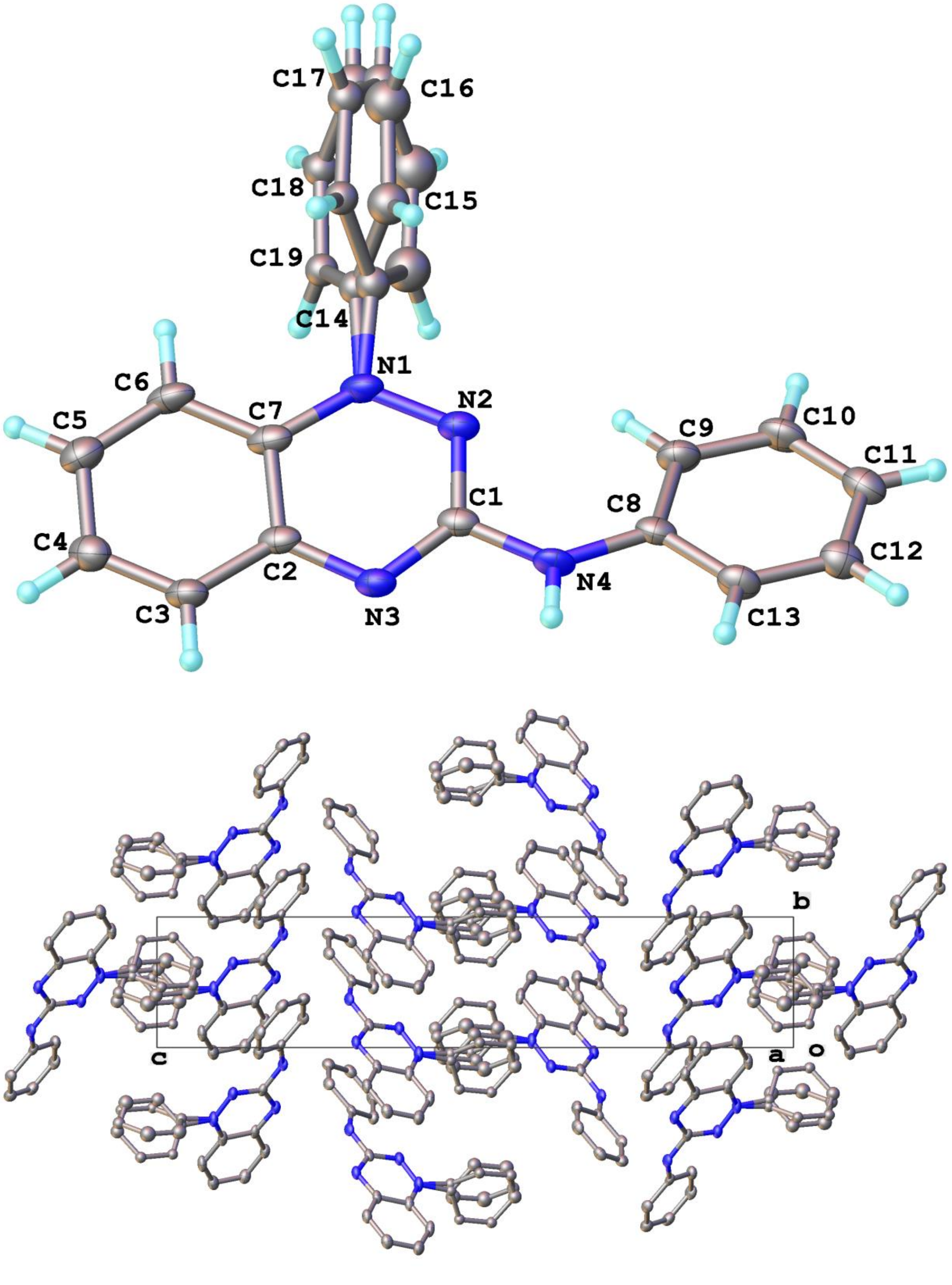

Supplementary Figure 5. Partially disordered molecule **4** and packing of molecules **4** in the crystal showing slipped stacking along b-axis.

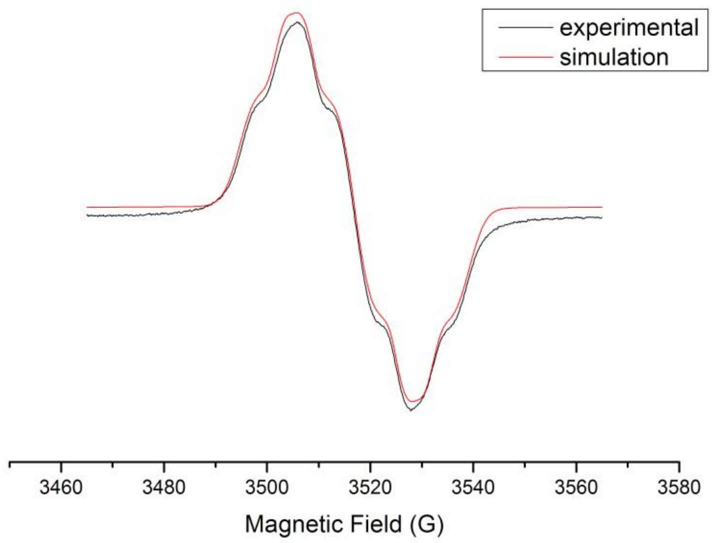

Supplementary Figure 6. EPR spectrum of **4** (a_{N1} = 7.84 G, a_{N2} = 4.92 G, a_{N3} = 4.88 G).

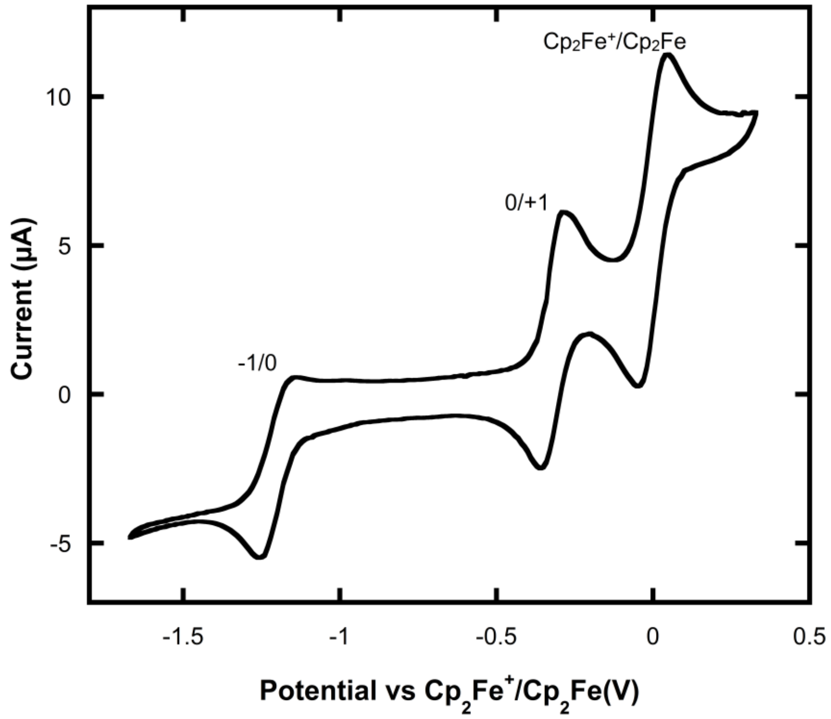

Supplementary Figure 7. Cyclic voltammogram of **4**.

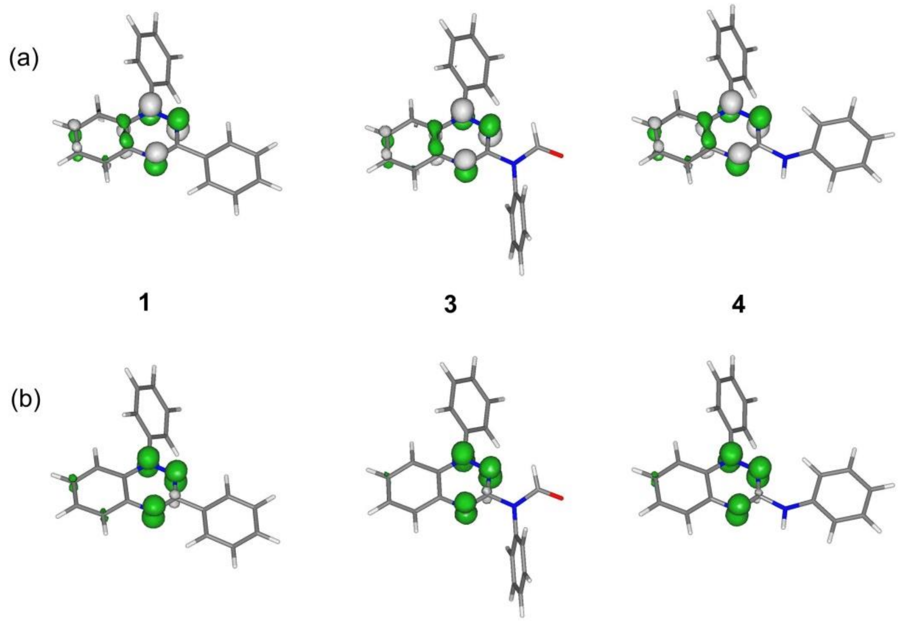

Supplementary Figure 8. Comparison of the SOMO (a) and spin densities (b) in radicals **1**, **3** and **4**.

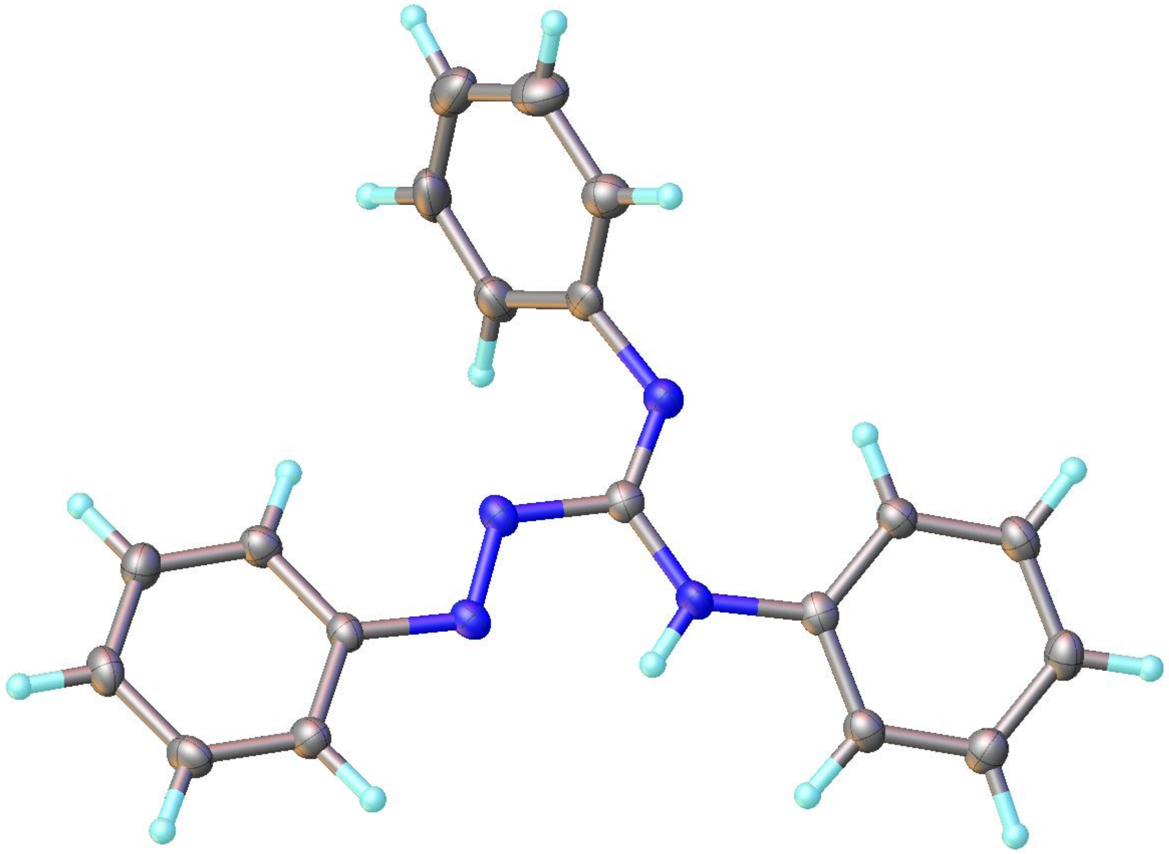

Supplementary Figure 9. Crystal structure of anilino-triazabutadiene **8**.

(**a**)

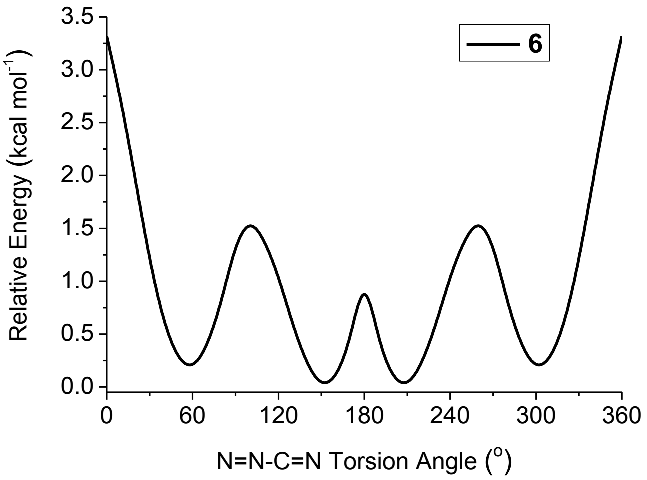

(**b**)

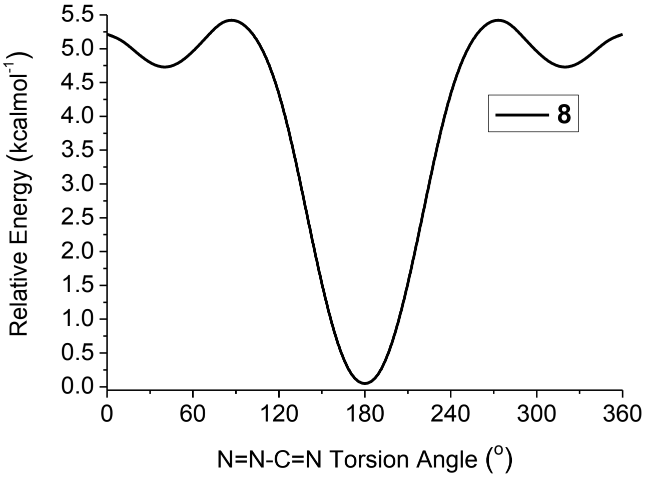

(**c**)

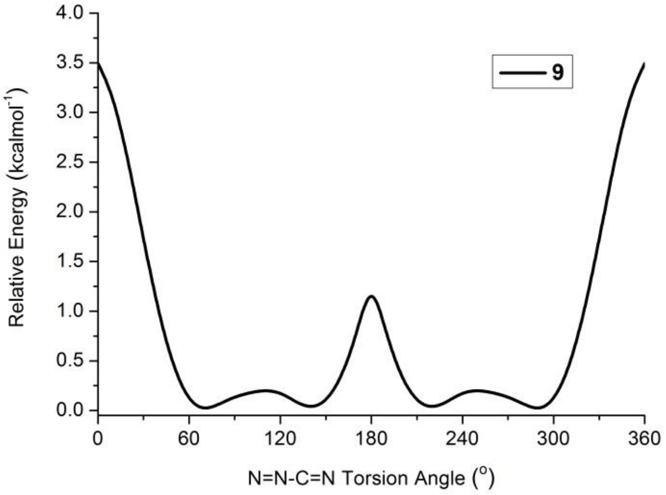

Supplementary Figure 10. Relative rotational energy profiles for (**a**) **6**, (**b**) **8** and (**c**) **9** where the *s-trans* and *s-cis* conformers correspond to 0° and 180° N=N–C=N torsion angles, respectively. Rotational energy barriers for **6**, **8** and **9** are 3.3, 5.4 and 3.5 kcal mol^{-1}.

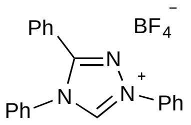

10

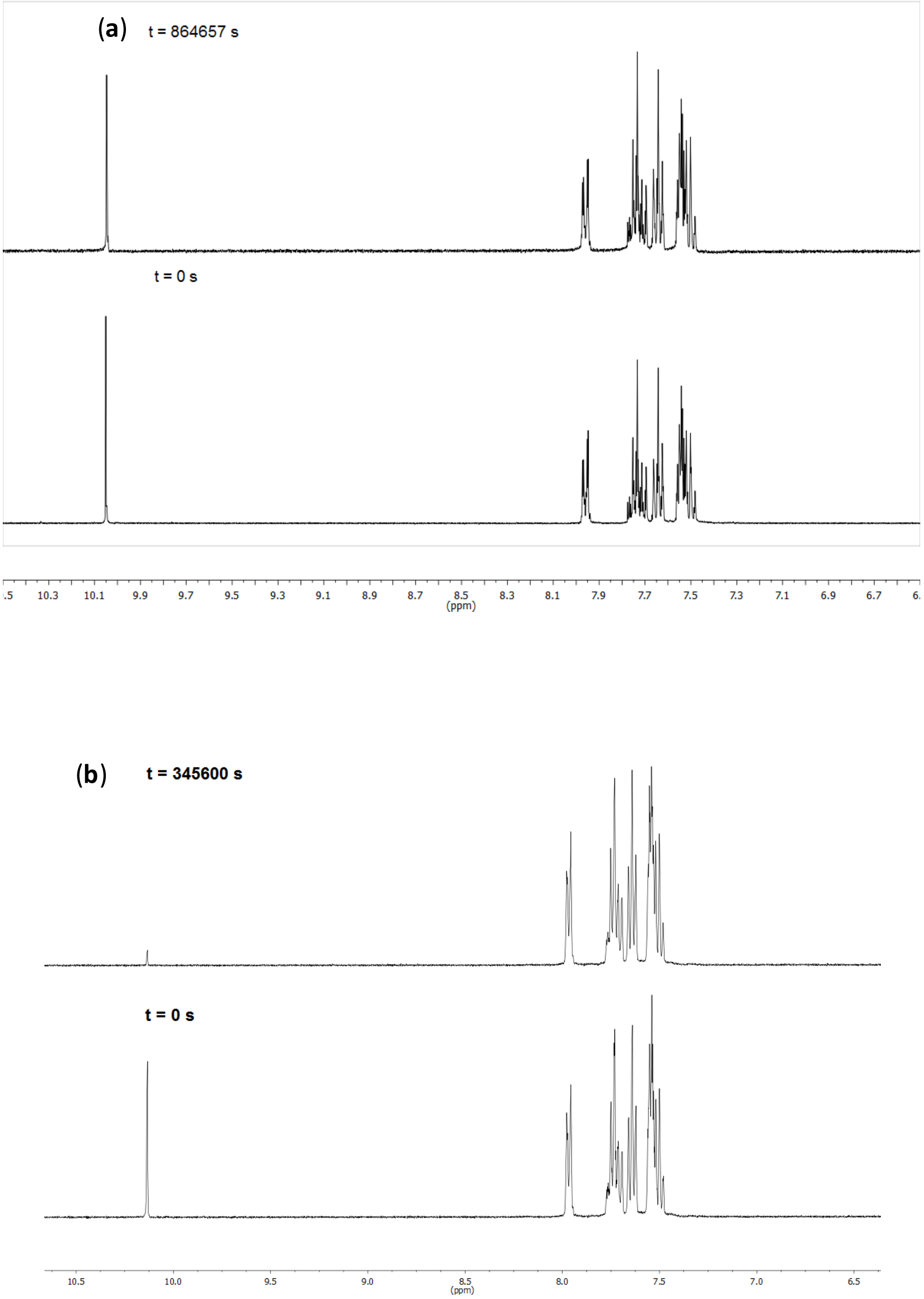

Supplementary Figure 11. ^{1}H NMR spectra of 1,2,4-triphenyltriazolium tetrafluoroborate **10** (**a**) in CD_3CN showing no change over 10 days and (**b**) in 99:1 CD_3CN:D_2O showing only exchange of the C(5)-hydrogen for deuterium and no other reactions over 4 days.

(**a**)

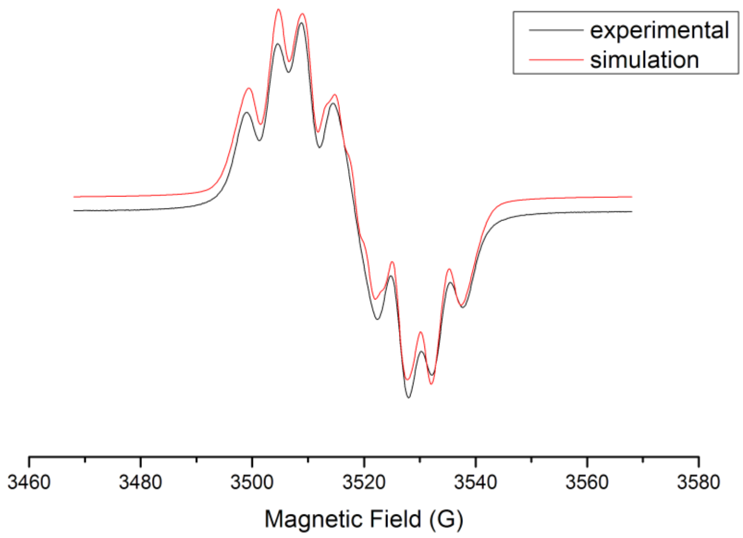
experimental
simulation
3460
3480
3500
3520
3540
3560
3580
Magnetic Field (G)

(**b**)

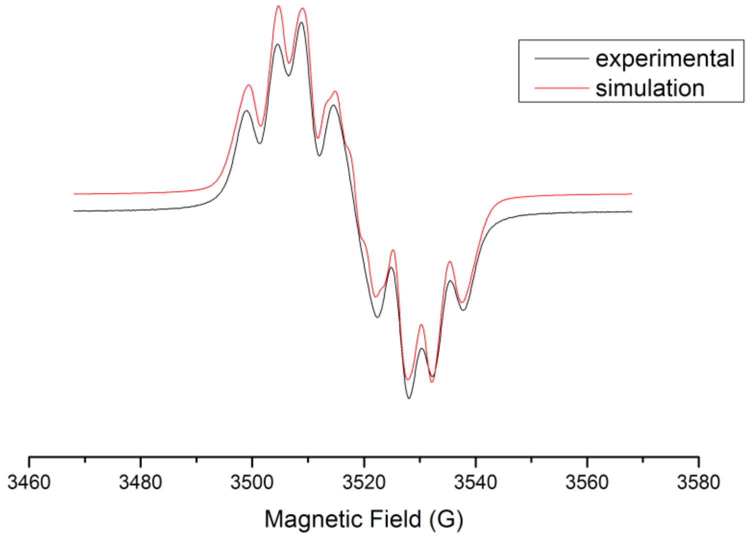
experimental
simulation
3460
3480
3500
3520
3540
3560
3580
Magnetic Field (G)

(**c**)

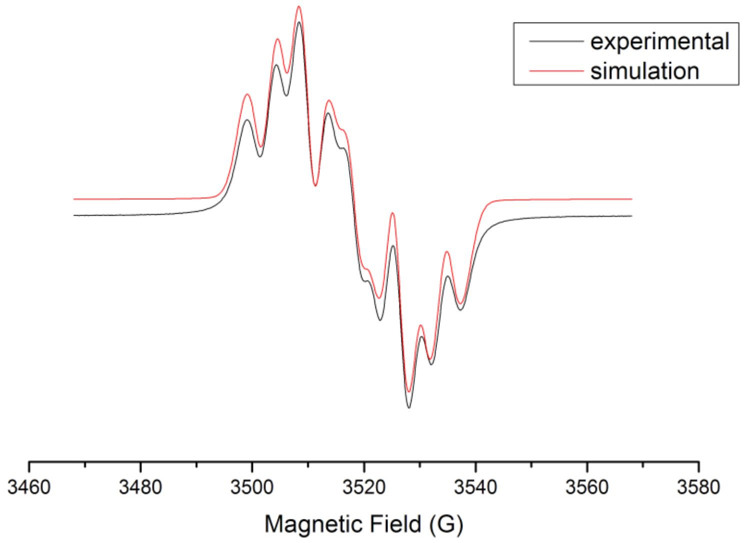

(**d**)

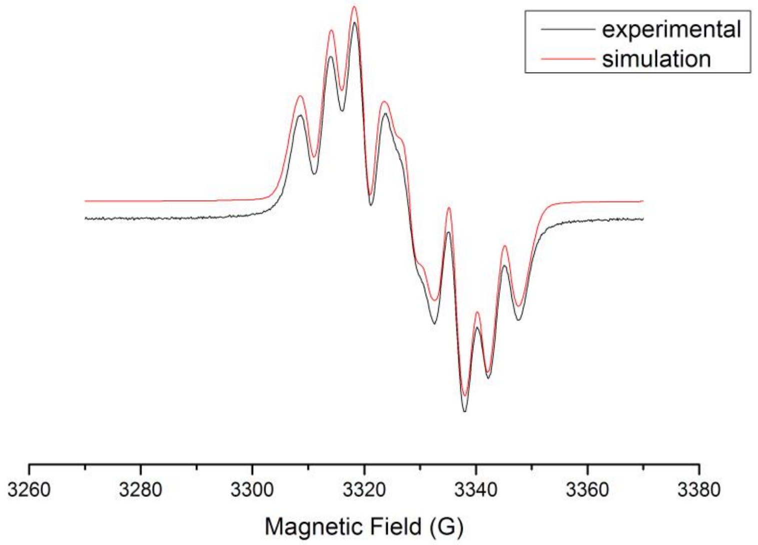

Supplementary Figure 12. Experimental and simulated EPR spectra at room temperature in toluene of (**a**) **14** (a_{N1} = 7.84 G, a_{N2} = 4.84 G, a_{N3} = 4.94 G), (**b**) **15** (a_{N1} = 7.79 G, a_{N2} = 4.90 G, a_{N3} = 4.84 G), (**c**) **16a** (a_{N1} = 7.99 G, a_{N2} = 4.89 G, a_{N3} = 4.54 G) and (**d**) **16b** (a_{N1} = 8.06 G, a_{N2} = 4.96 G, a_{N3} = 4.79 G).

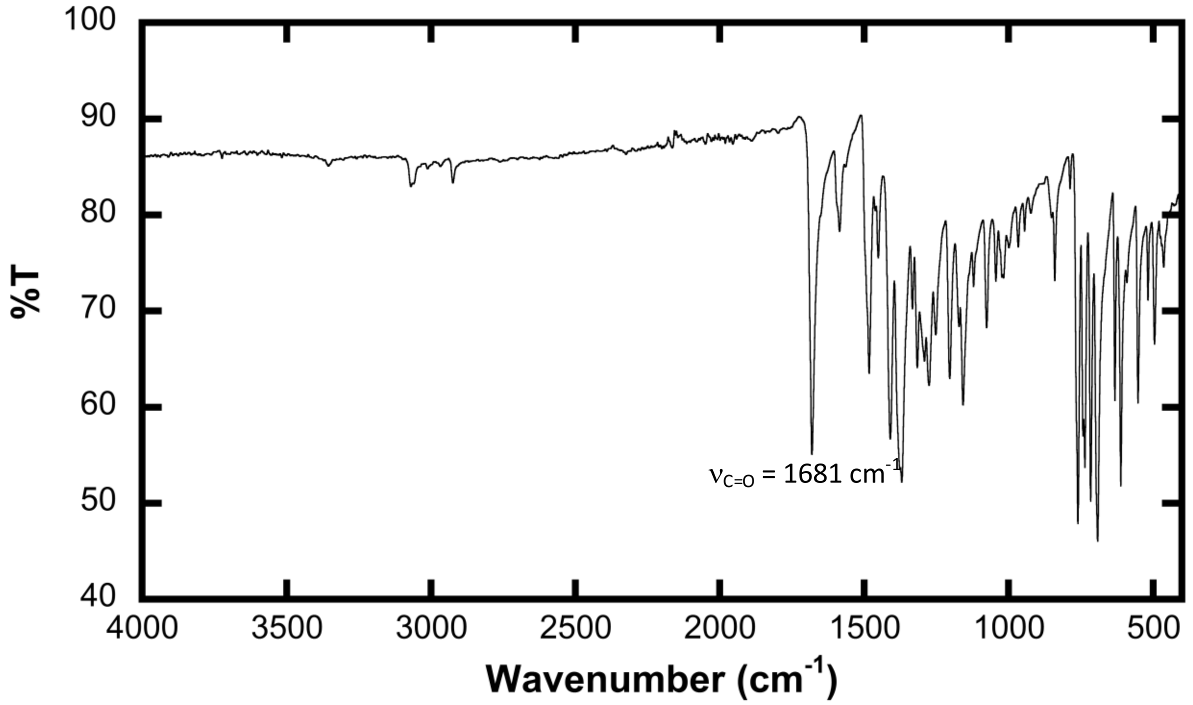

Supplementary Figure 13 IR spectrum of **3**.

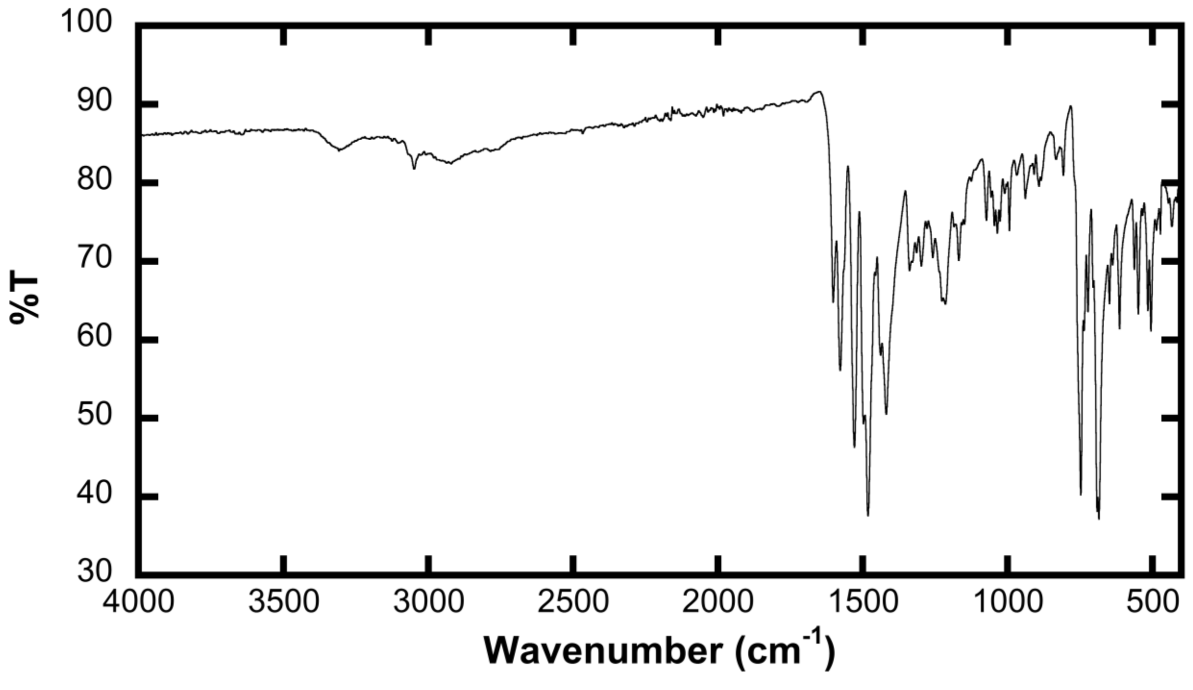

Supplementary Figure 14 IR spectrum of **4**.

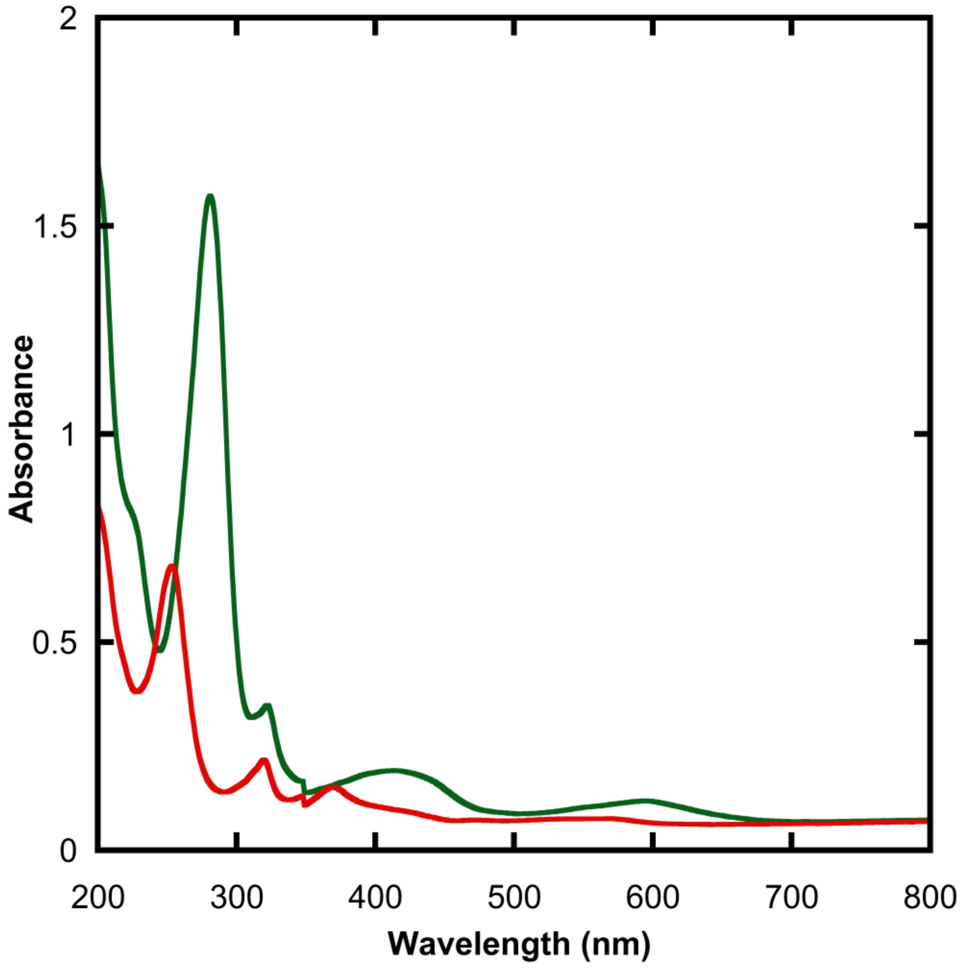

Supplementary Figure 15 UV visible spectra for **3** (—, 0.02mM) and **4** (—, 0.05mM) in acetonitrile at 25 °C.

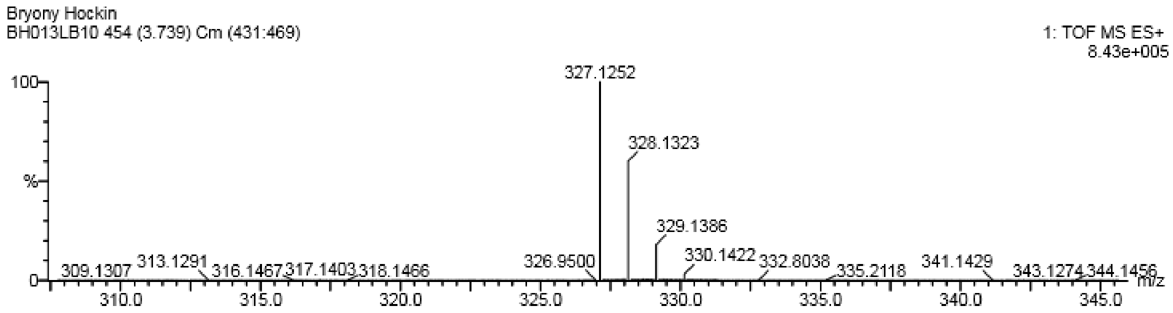

Supplementary Figure 16 Mass spectrum of **3**.

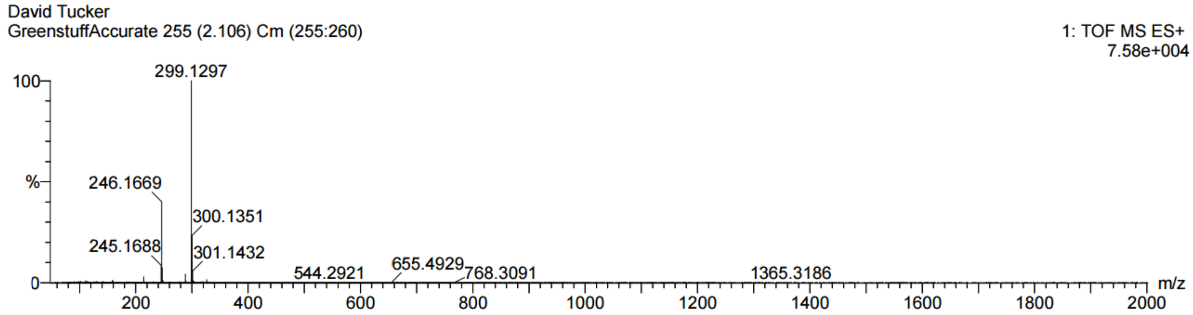

Supplementary Figure 17 Mass spectrum of **4**.

York - Chemistry - Mass Spectrometry Service Report

Green one

Analysis Information

Acquisition Date 03/02/2014 10:10:30

Analysis Filename	vc44797zl_000003.d
Method	KDH_MSService_ESI_MSMS
Submission Name	vc44797zl
Instrument	solariX
n/a	n/a

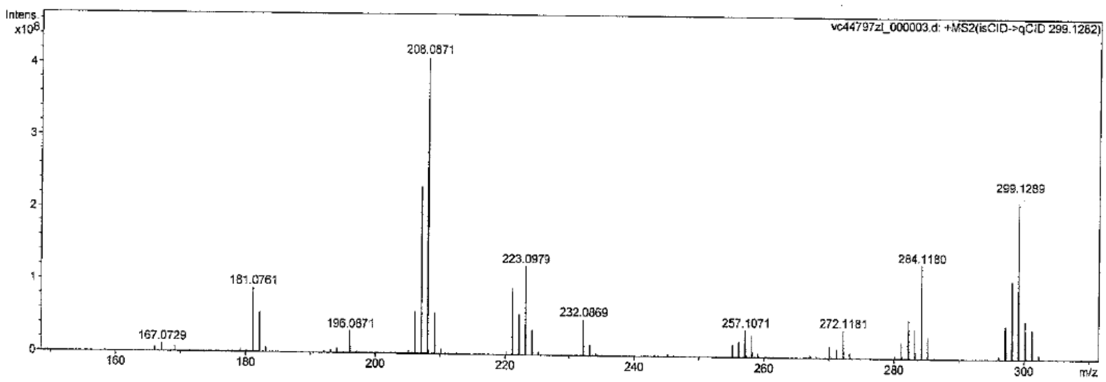

Meas. m/z	#	Formula	m/z	err [ppm]	err [mDa]	mSigma	Mean err [ppm]
181.0761	1	C 13 H 11 N	181.0886	69.2	12.5	83.5	69.2
196.0871	1	C 13 H 12 N 2	196.0995	63.4	12.4	85.6	63.4
208.0871	1	C 13 H 10 N 3	208.0869	-0.9	-0.2	8.1	-0.9
223.0979	1	C 13 H 11 N 4	223.0978	-0.2	-0.0	2.0	-0.4
284.1180	1	C 19 H 14 N 3	284.1182	0.7	0.2	11.3	0.7
299.1289	1	C 19 H 15 N 4	299.1291	0.7	0.2	12.5	0.7

Supplementary Figure 18 MS-MS analysis of 4.

Single Mass Analysis
Tolerance = 5.0 mDa / DBE: min = -1.5, max = 50.0
Element prediction: Off
Number of isotope peaks used for i-FIT = 3

Monoisotopic Mass, Even Electron Ions
967 formula(e) evaluated with 15 results within limits (up to 500 best isotopic matches for each mass)
Elements Used:
C: 0-75 H: 0-85 10B: 0-1 N: 0-8 O: 0-7 Na: 0-1
David Tucker

27-Sep-2016
Red_triazabutadiene 503 (4.230) Cm (503:511)
1: TOF MS ES+
4.20e+004

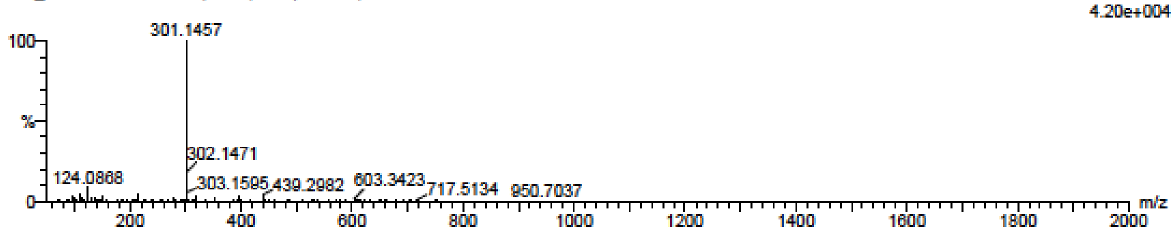

Minimum:				-1.5			
Maximum:		5.0	5.0	50.0			
Mass	Calc. Mass	mDa	PPM	DBE	i-FIT	i-FIT (Norm)	Formula
301.1457	301.1440	1.7	5.6	8.5	690.8	1.6	C18 H21 O4
	301.1416	4.1	13.6	5.5	691.1	1.9	C16 H22 O4 Na
	301.1501	-4.4	-14.6	12.5	691.5	2.3	C18 H17 10B N3 O
	301.1477	-2.0	-6.6	9.5	691.5	2.4	C16 H18 10B N3 O Na
	301.1429	2.8	9.3	10.5	691.7	2.5	C17 H18 N4 Na
	301.1453	0.4	1.3	13.5	691.7	2.6	C19 H17 N4
	301.1413	4.4	14.6	9.5	691.8	2.6	C14 H17 N6 O2
	301.1461	-0.4	-1.3	8.5	691.9	2.7	C13 H17 10B N5 O3
	301.1447	1.0	3.3	3.5	692.1	2.9	C12 H21 10B N O7
	301.1437	2.0	6.6	5.5	692.7	3.5	C11 H18 10B N5 O3 Na
	301.1501	-4.4	-14.6	6.5	692.8	3.7	C11 H18 N8 O Na
	301.1488	-3.1	-10.3	1.5	692.9	3.8	C10 H22 N4 O5 Na
	301.1423	3.4	11.3	0.5	693.1	3.9	C10 H22 10B N O7 Na
	301.1421	3.6	12.0	4.5	693.5	4.3	C8 H17 10B N7 O5
	301.1472	-1.5	-5.0	0.5	693.7	4.5	C7 H21 N6 O7

Supplementary Figure 19 Mass spectrum of **8**.

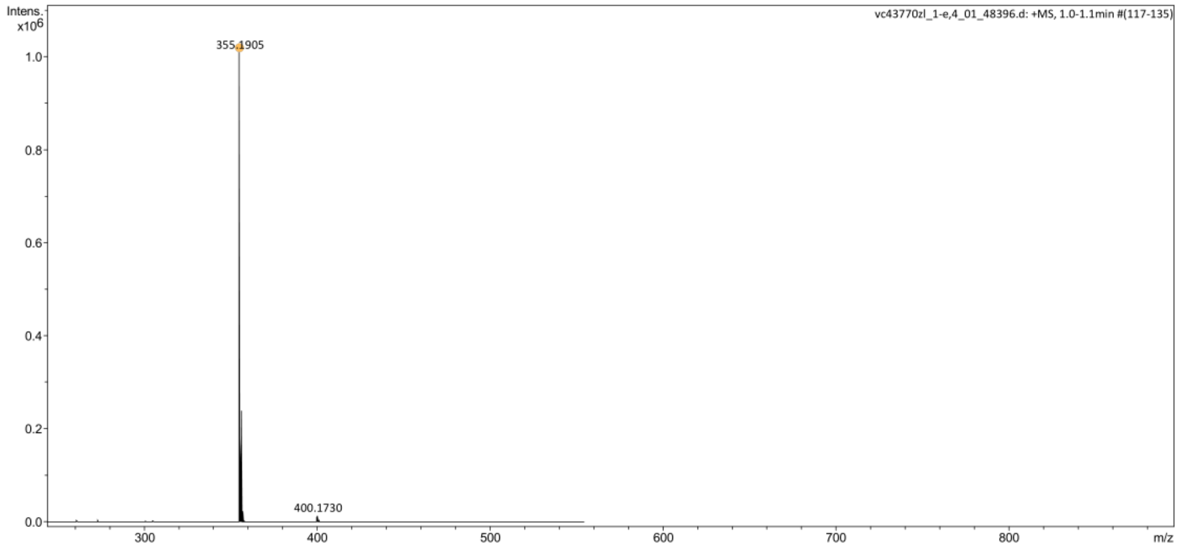

Supplementary Figure 20 Mass spectrum of **11**.

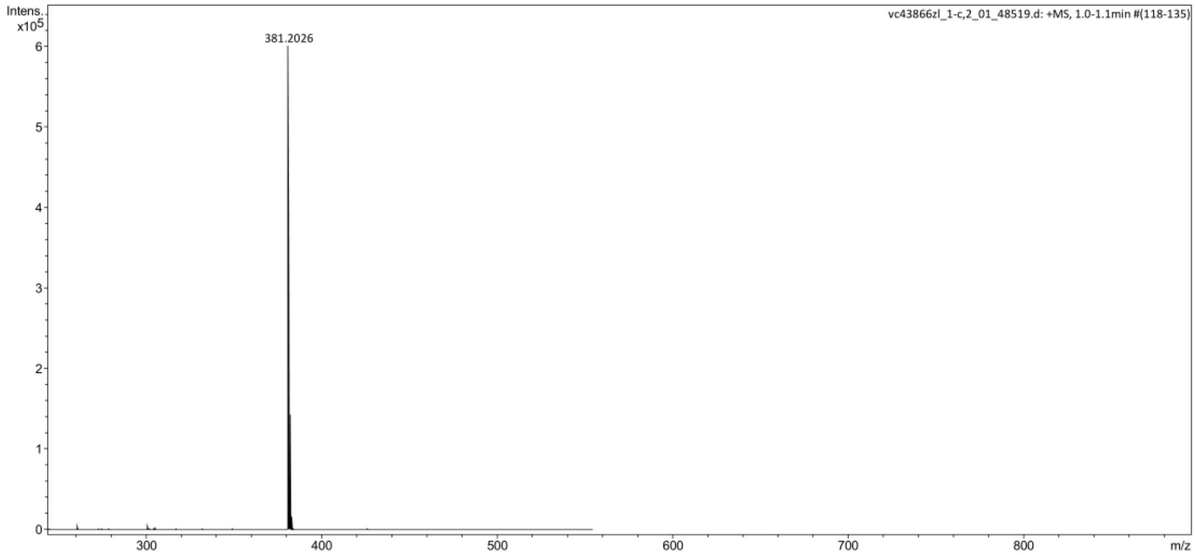

Supplementary Figure 21 Mass spectrum of **12**.

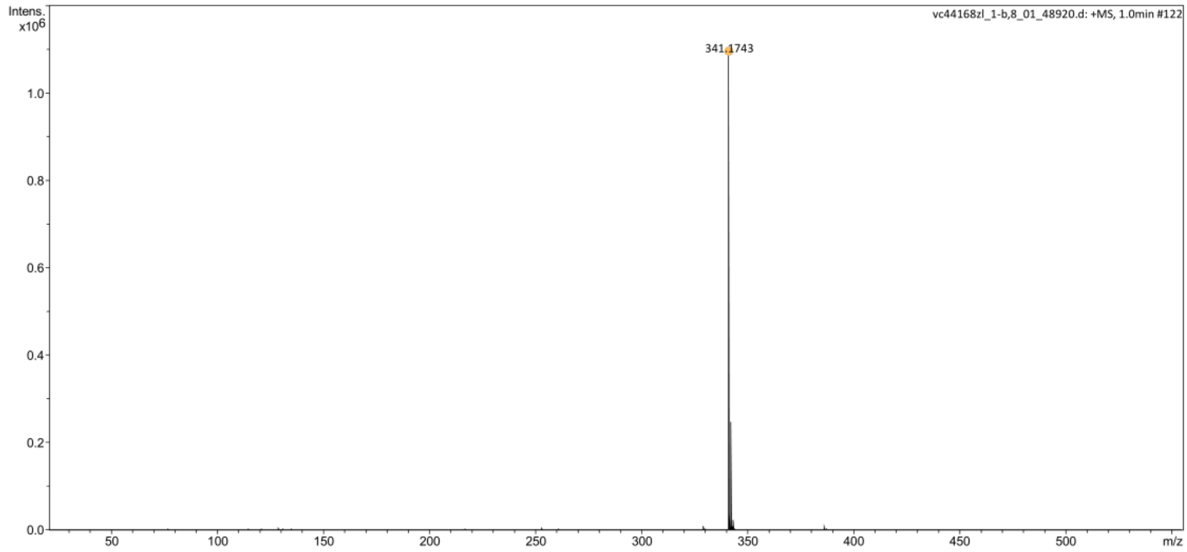

Supplementary Figure 22 Mass spectrum of **13a.**

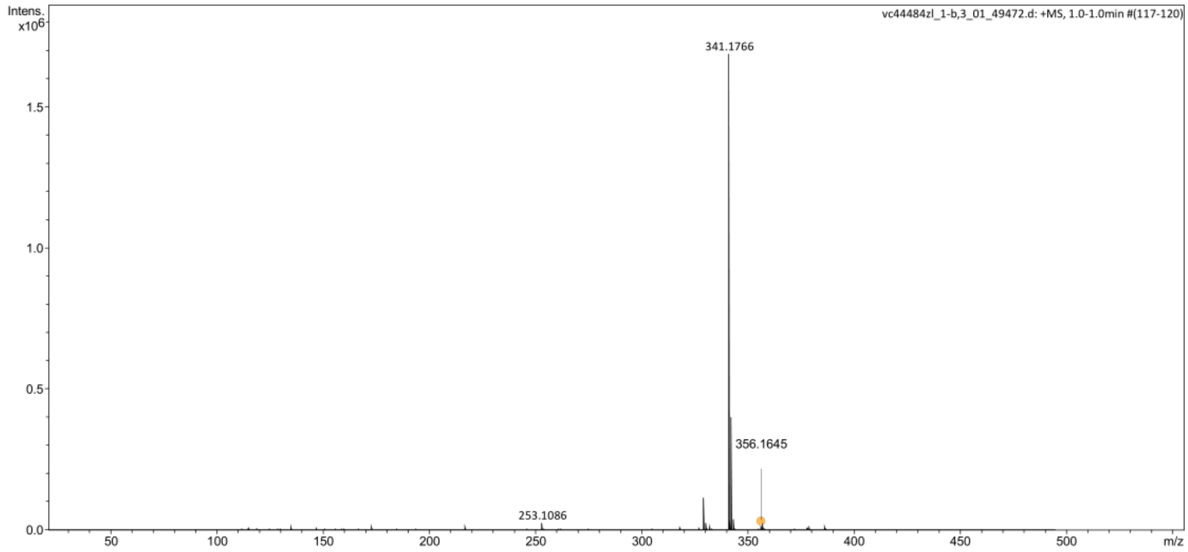

Supplementary Figure 23 Mass spectrum of **13b.**

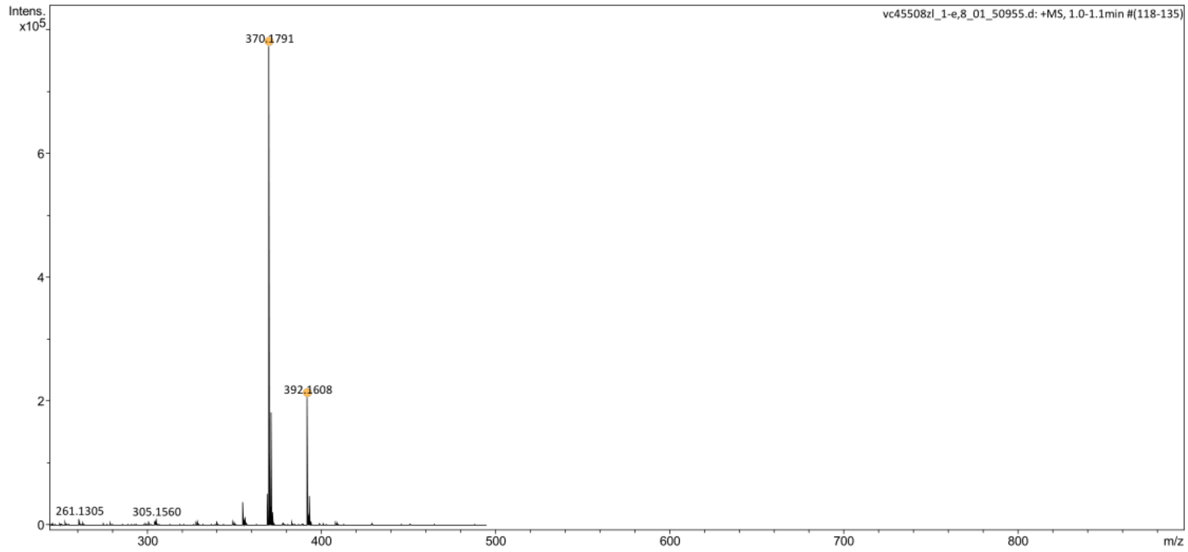

Supplementary Figure 24 Mass spectrum of **14.**

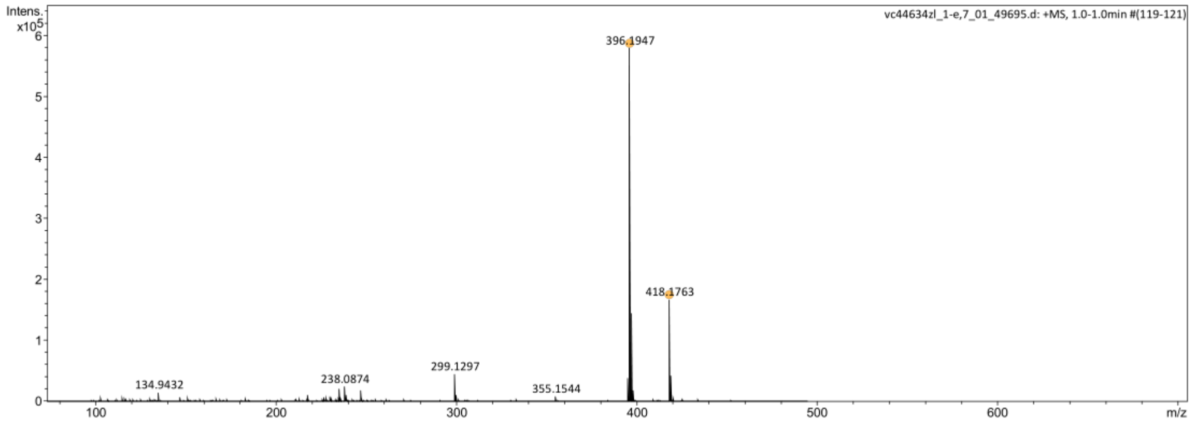

Supplementary Figure 25 Mass spectrum of **15.**

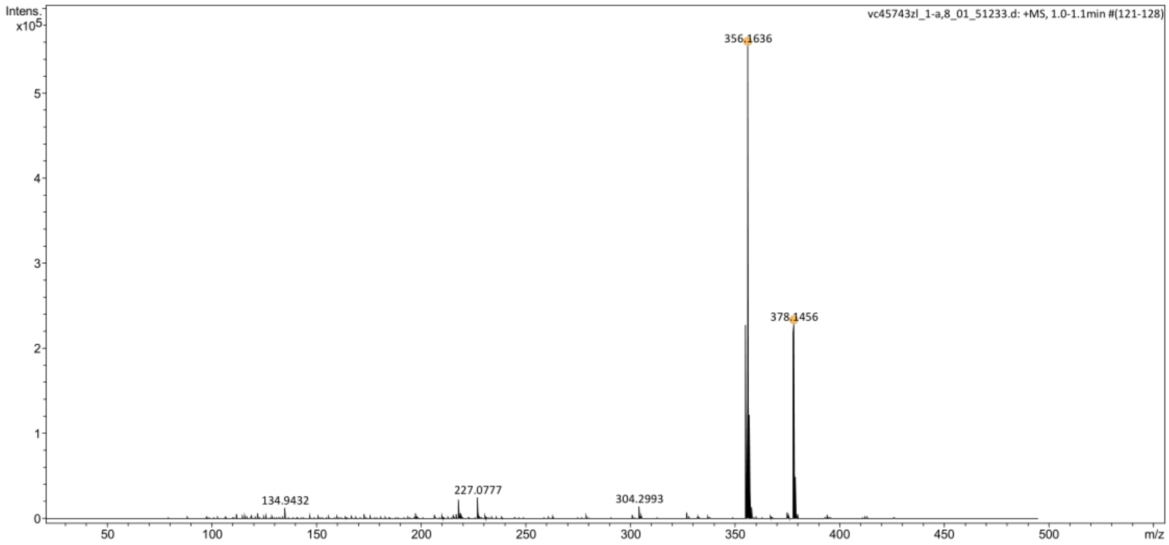

Supplementary Figure 26 Mass spectrum of **16a.**

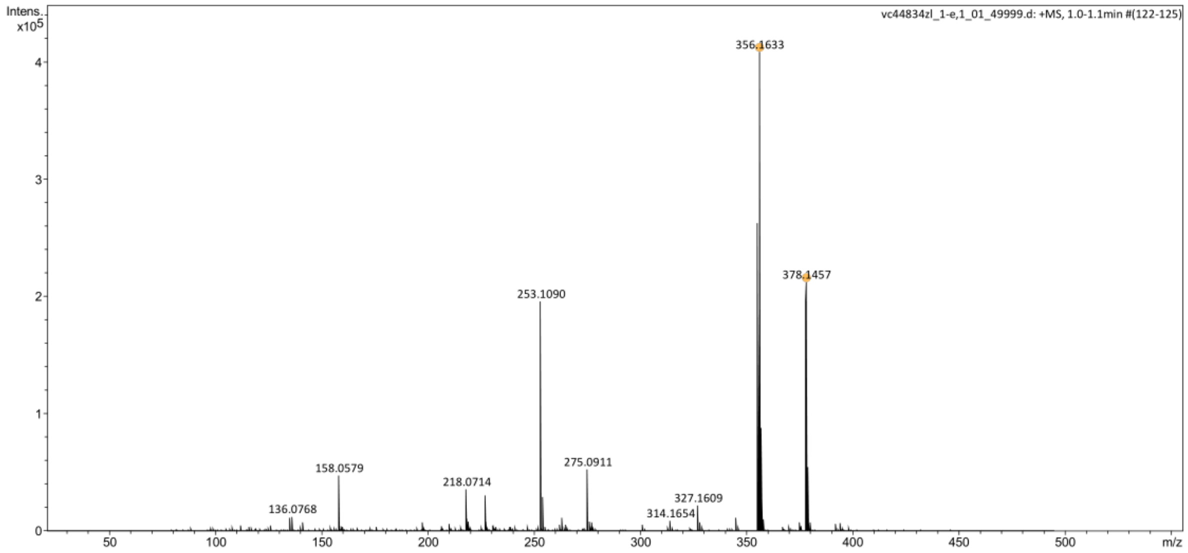

Supplementary Figure 27 Mass spectrum of **16b.**

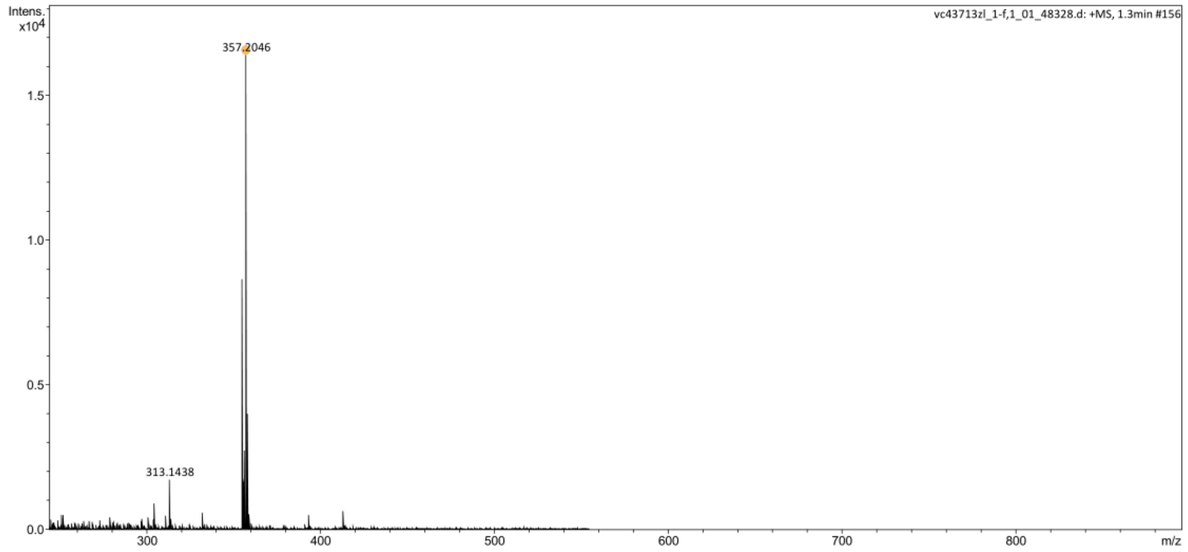

Supplementary Figure 28 Mass spectrum of **SI-4.**

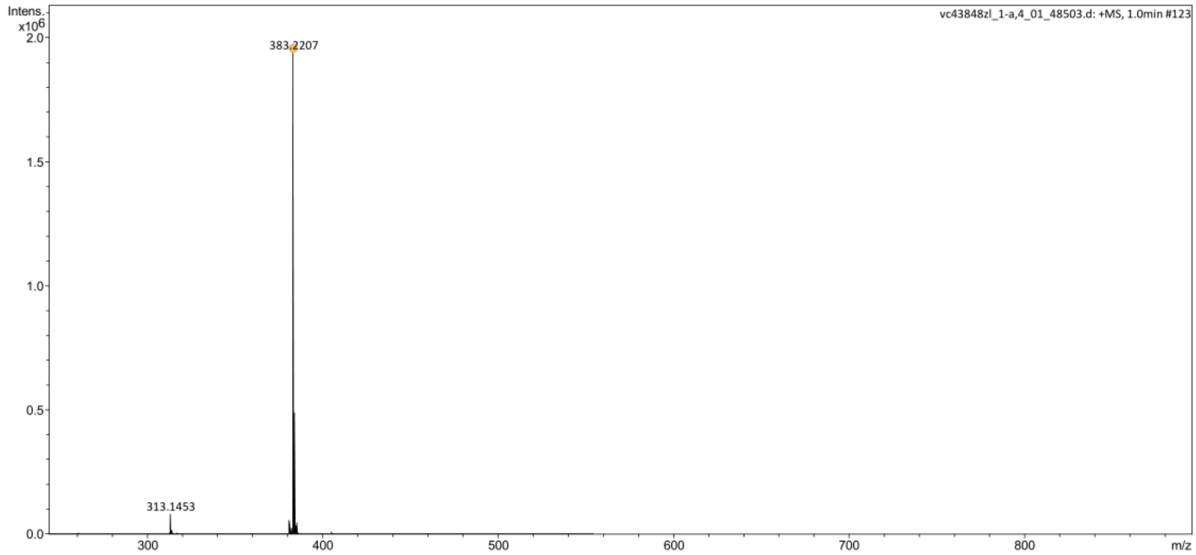

Supplementary Figure 29 Mass spectrum of **SI-5.**

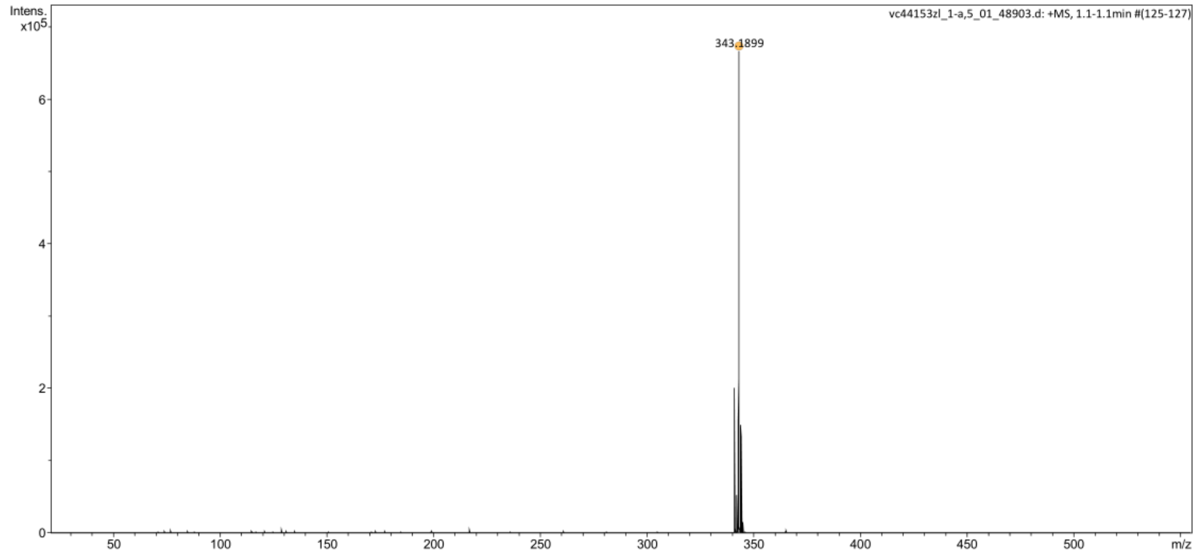

Supplementary Figure 30 Mass spectrum of **SI-6.**

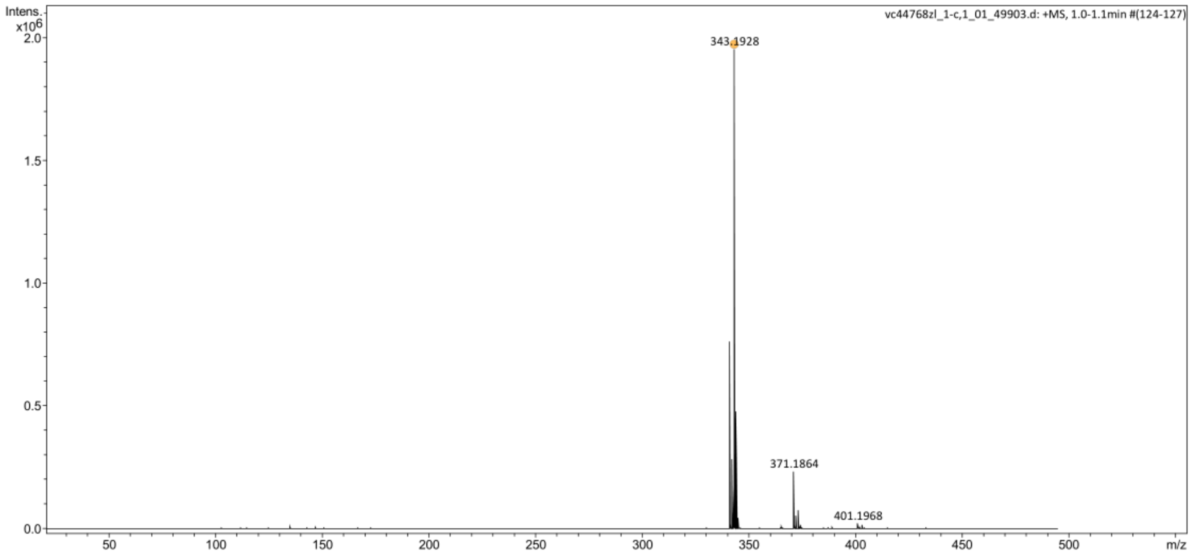

Supplementary Figure 31 Mass spectrum of **SI-7.**

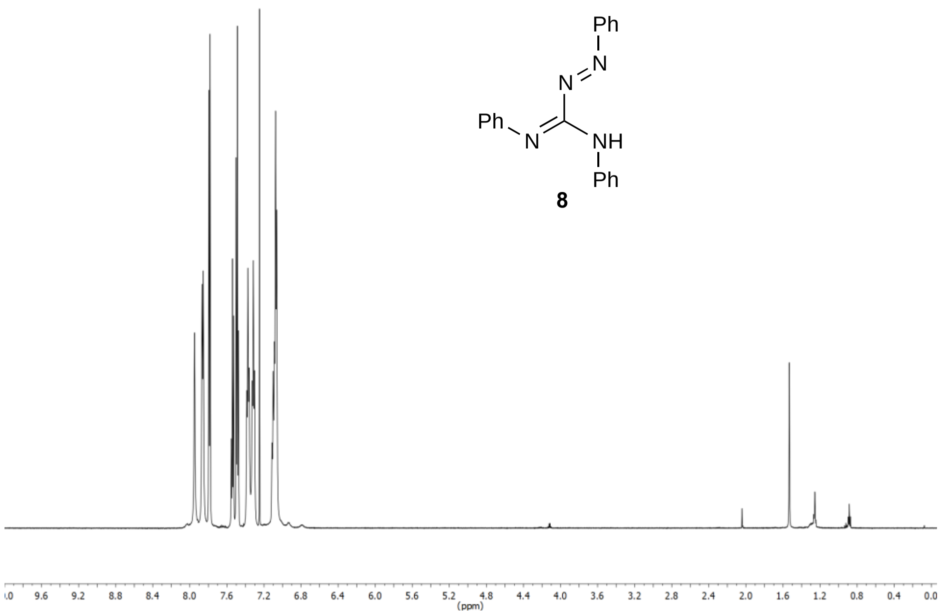

Supplementary Figure 32. ^{1}H NMR spectrum (600MHz) of **8** in $CDCl_3$.

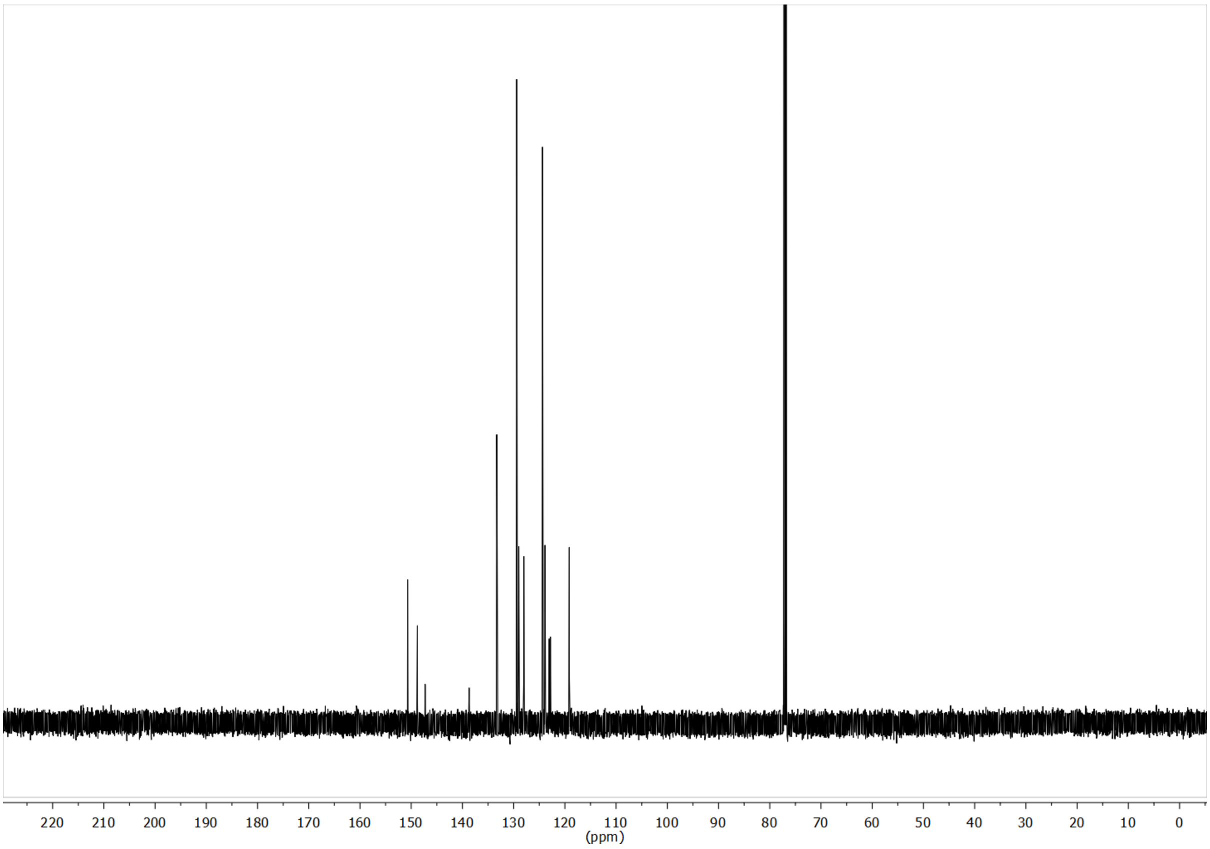

Supplementary Figure 33. ^{13}C NMR spectrum (150 MHz) of **8** in $CDCl_3$.

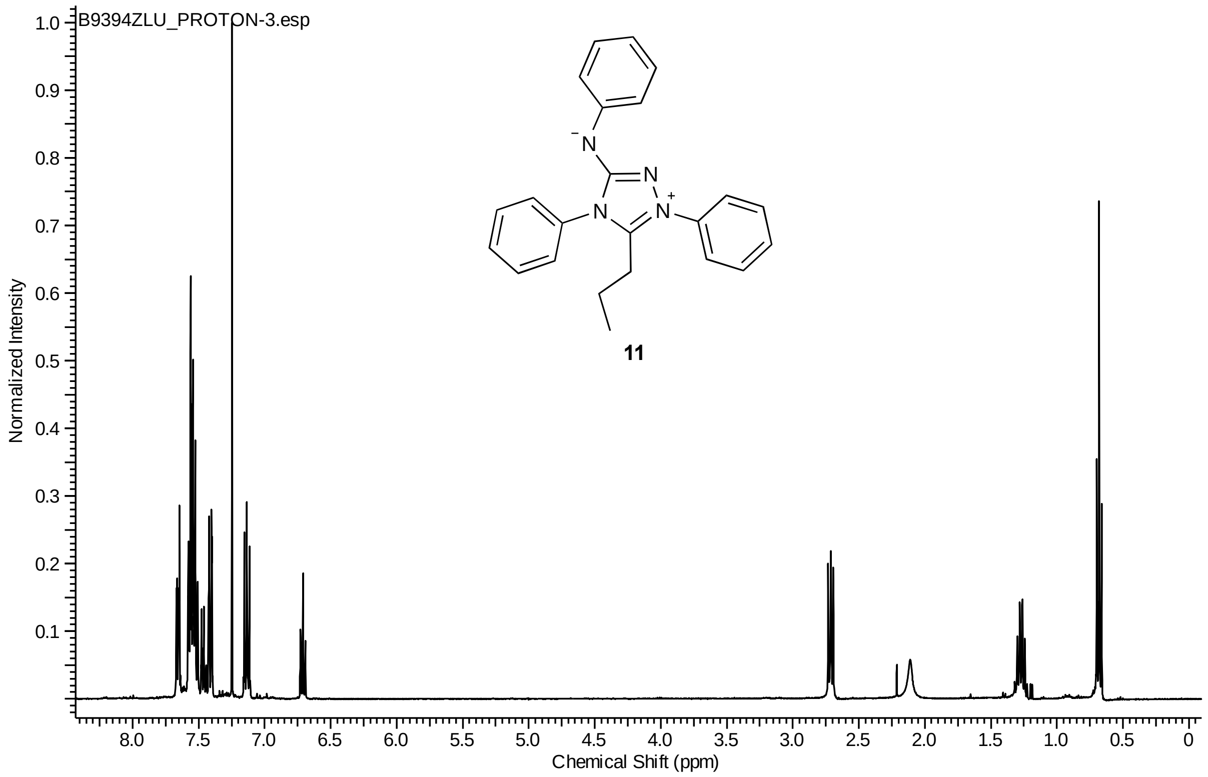

Supplementary Figure 34. ^{1}H NMR spectrum (400MHz) of **11** in $CDCl_3$.

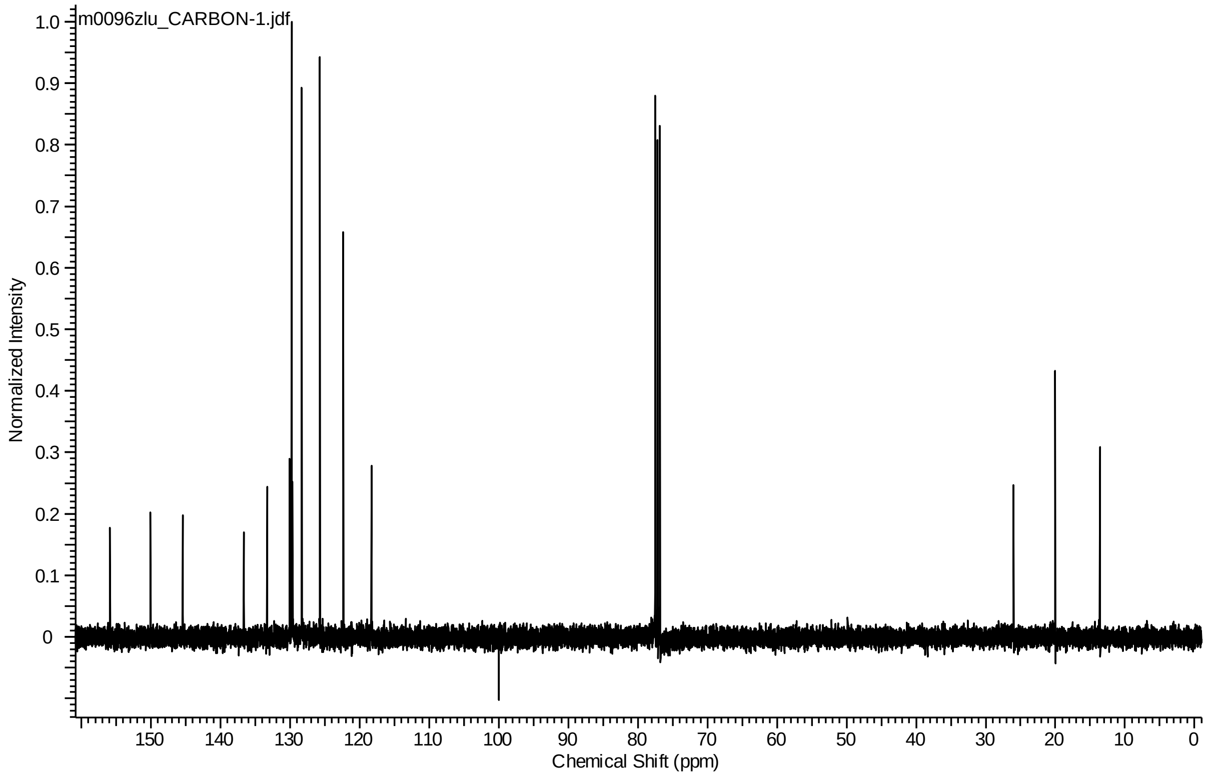

Supplementary Figure 35. ^{13}C NMR spectrum (100 MHz) of **11** in $CDCl_3$.

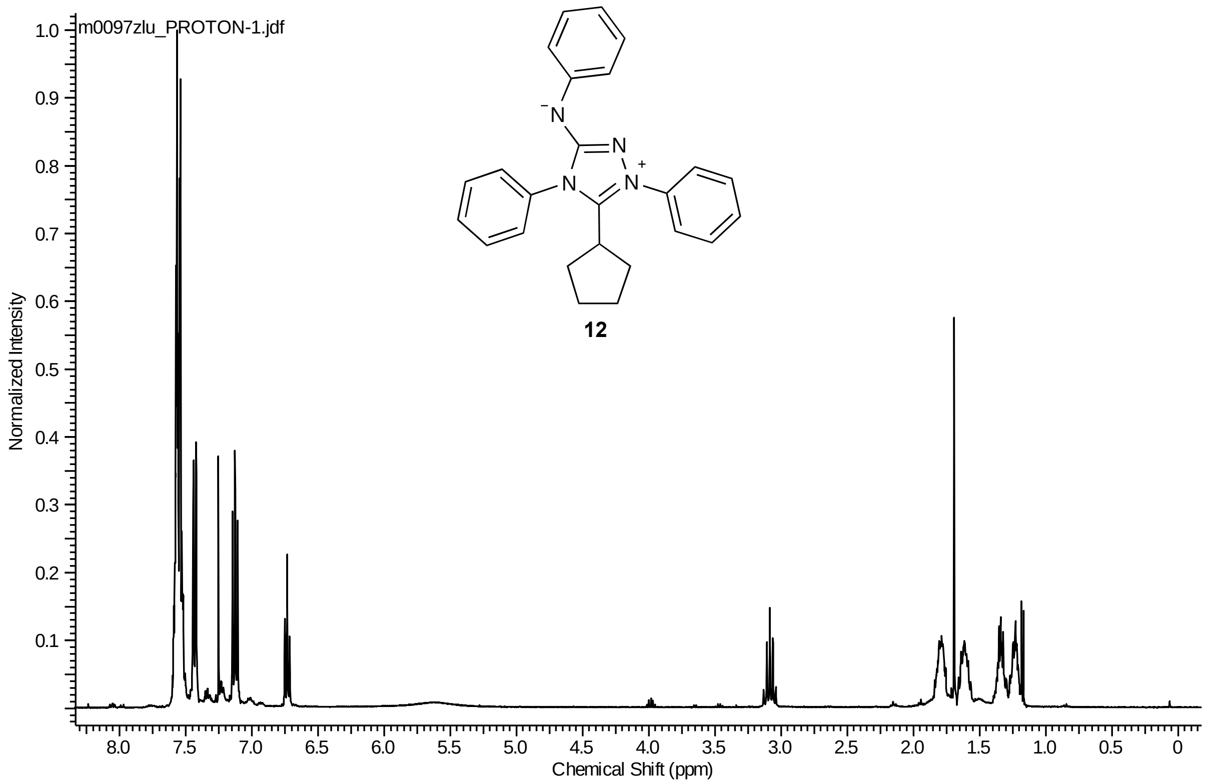

Supplementary Figure 36. ^{1}H NMR spectrum (400MHz) of **12** in $CDCl_3$.

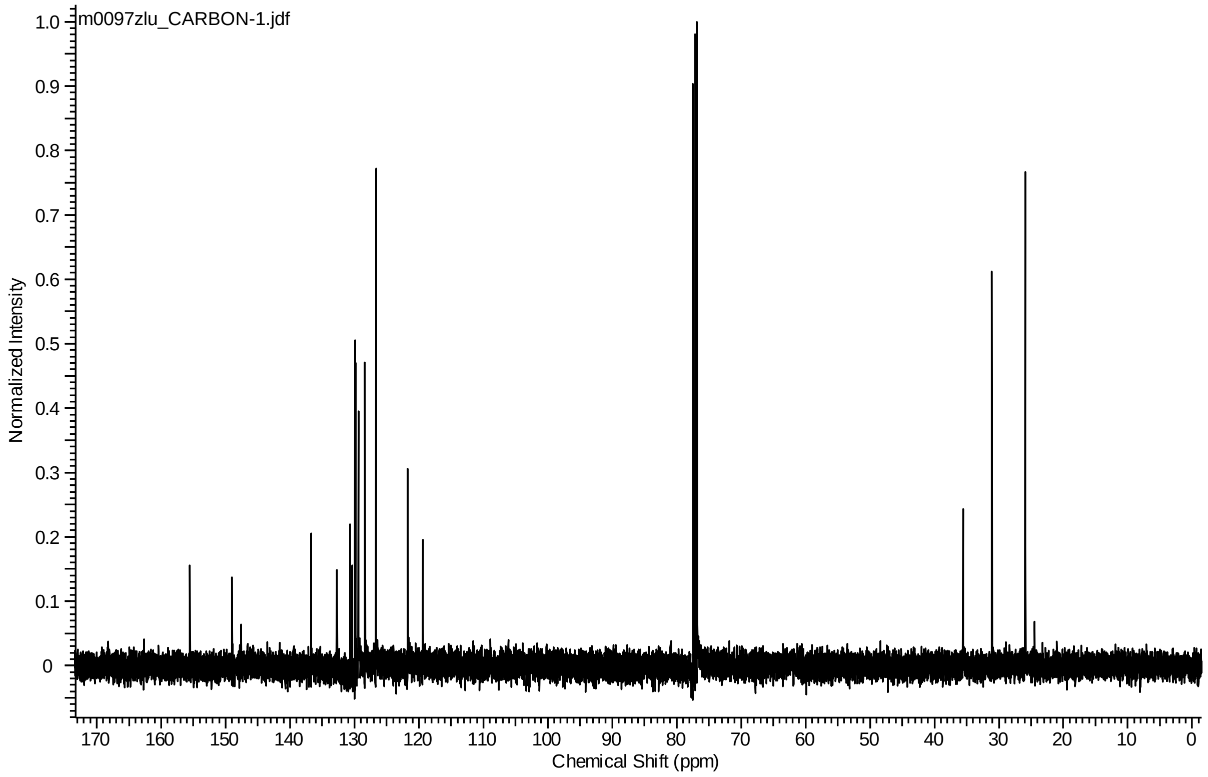

Supplementary Figure 37. ^{13}C NMR spectrum (100MHz) of **12** in $CDCl_3$.

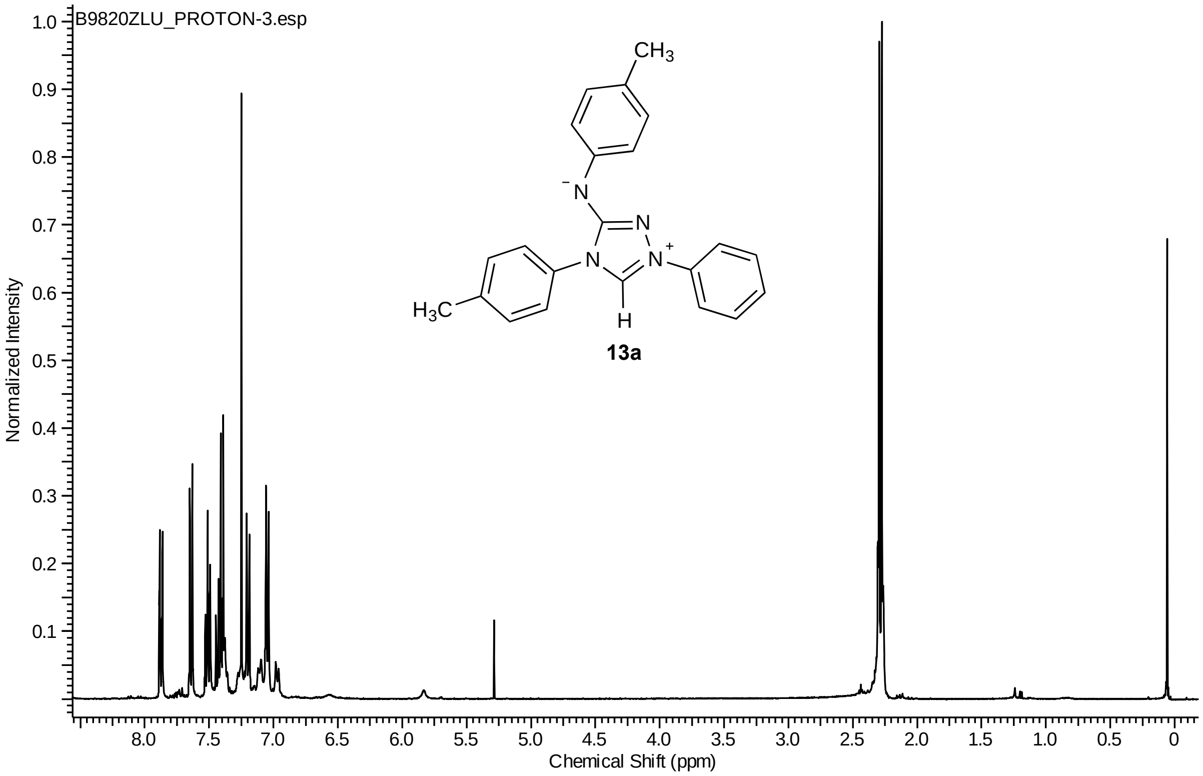

Supplementary Figure 38. ^{1}H NMR spectrum (400MHz) of **13a** in $CDCl_3$.

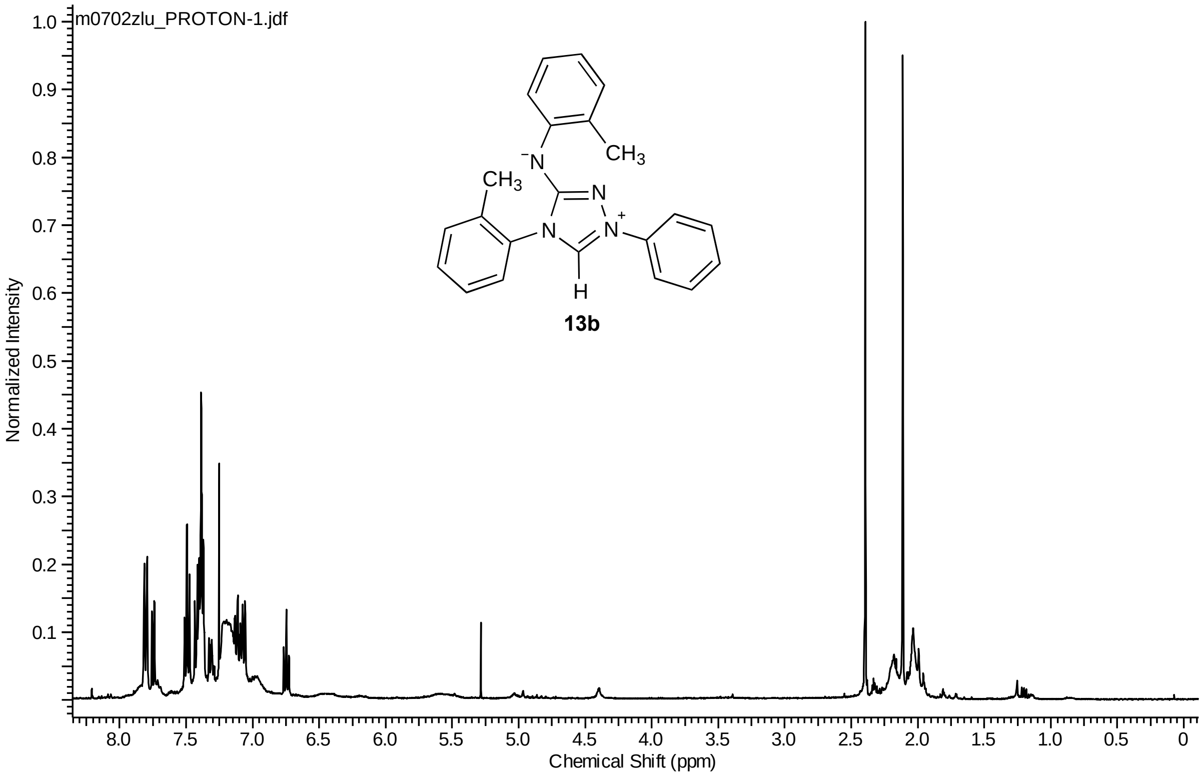

Supplementary Figure 39. ^{1}H NMR spectrum (400MHz) of **13b** in $CDCl_3$.

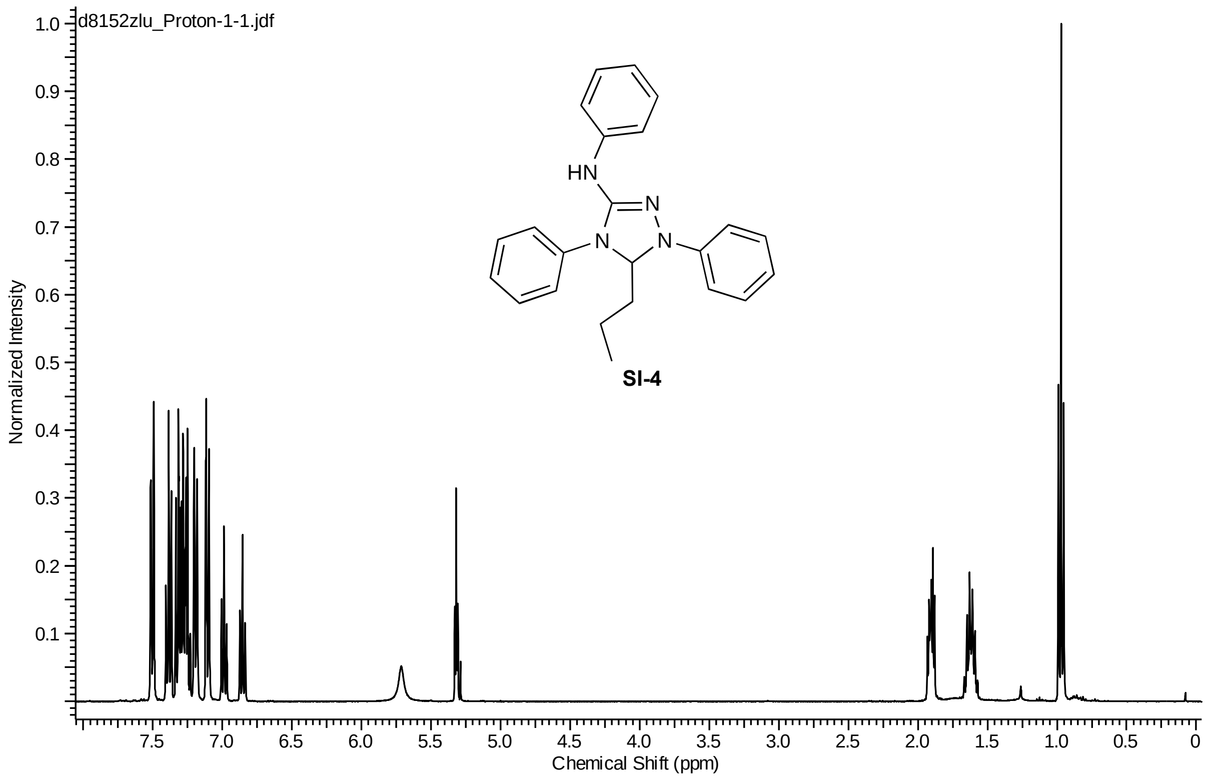

Supplementary Figure 40. ^{1}H NMR spectrum (400MHz) of **SI-4** in $CDCl_3$.

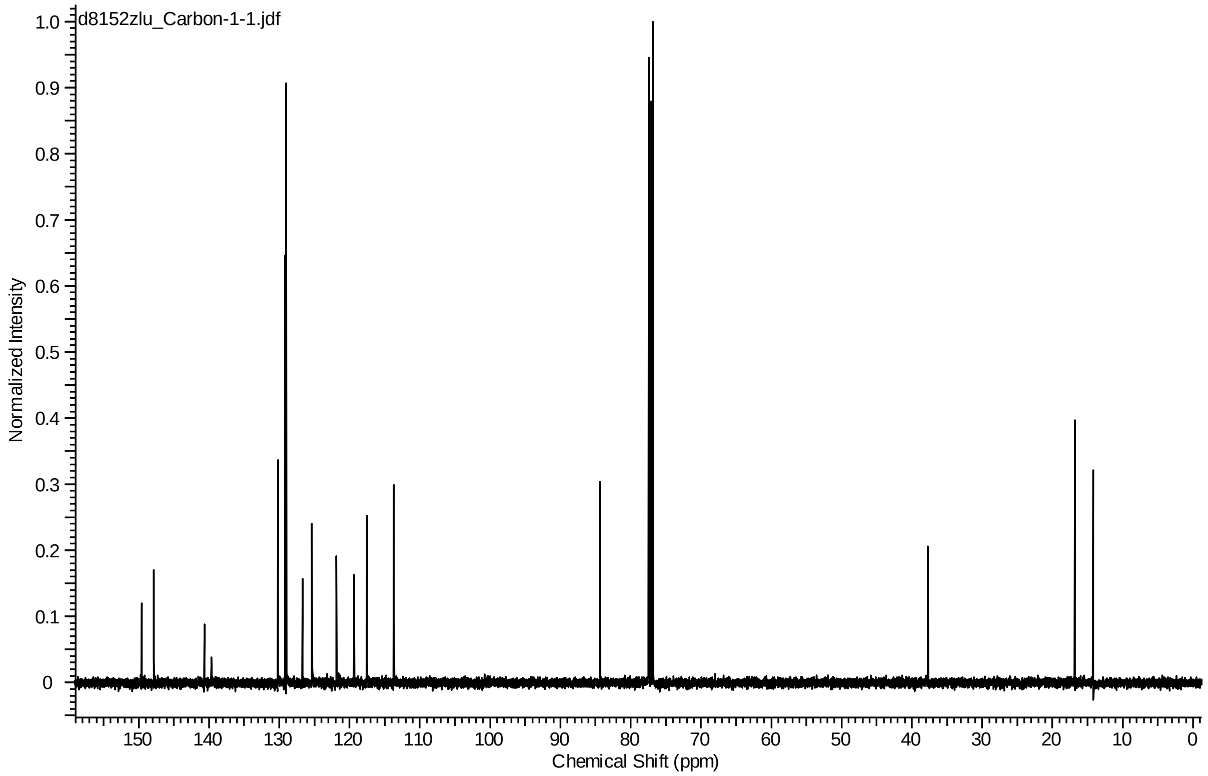

Supplementary Figure 41. ^{13}C NMR spectrum (100MHz) of **SI-4** in $CDCl_3$.

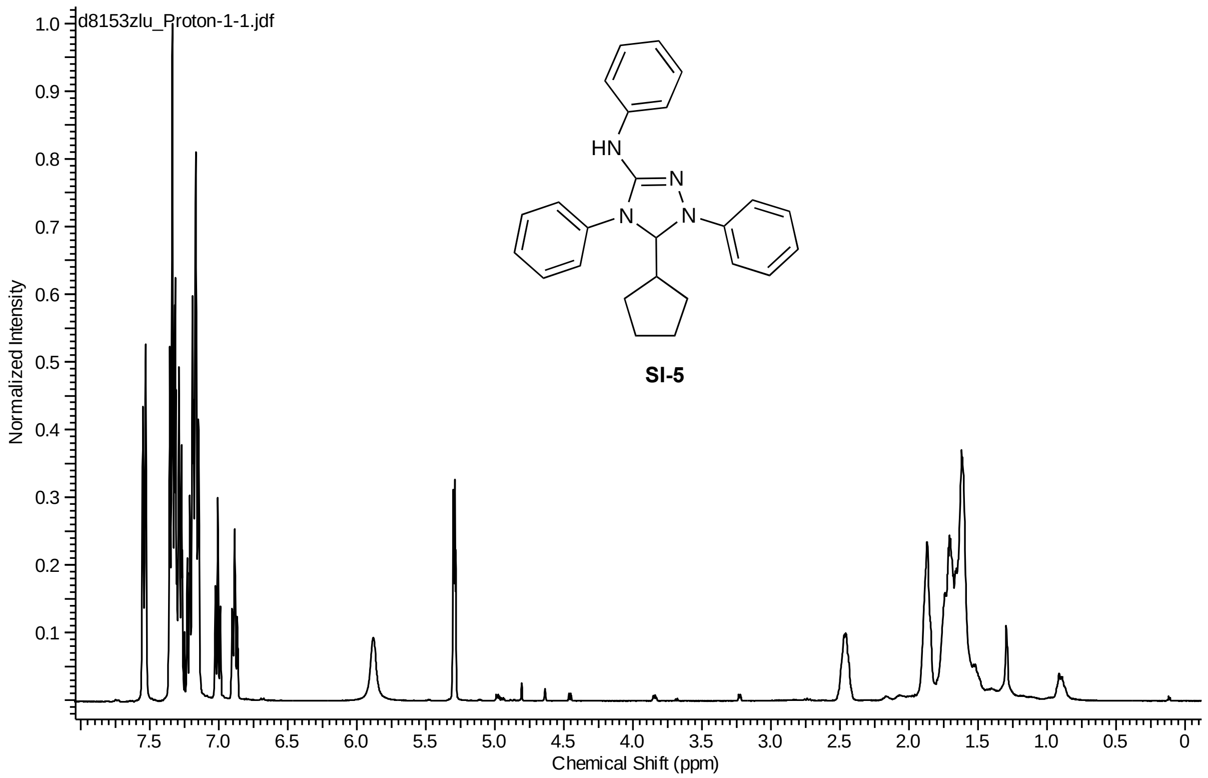

Supplementary Figure 42. ^{1}H NMR spectrum (400MHz) of **SI-5** in $CDCl_3$.

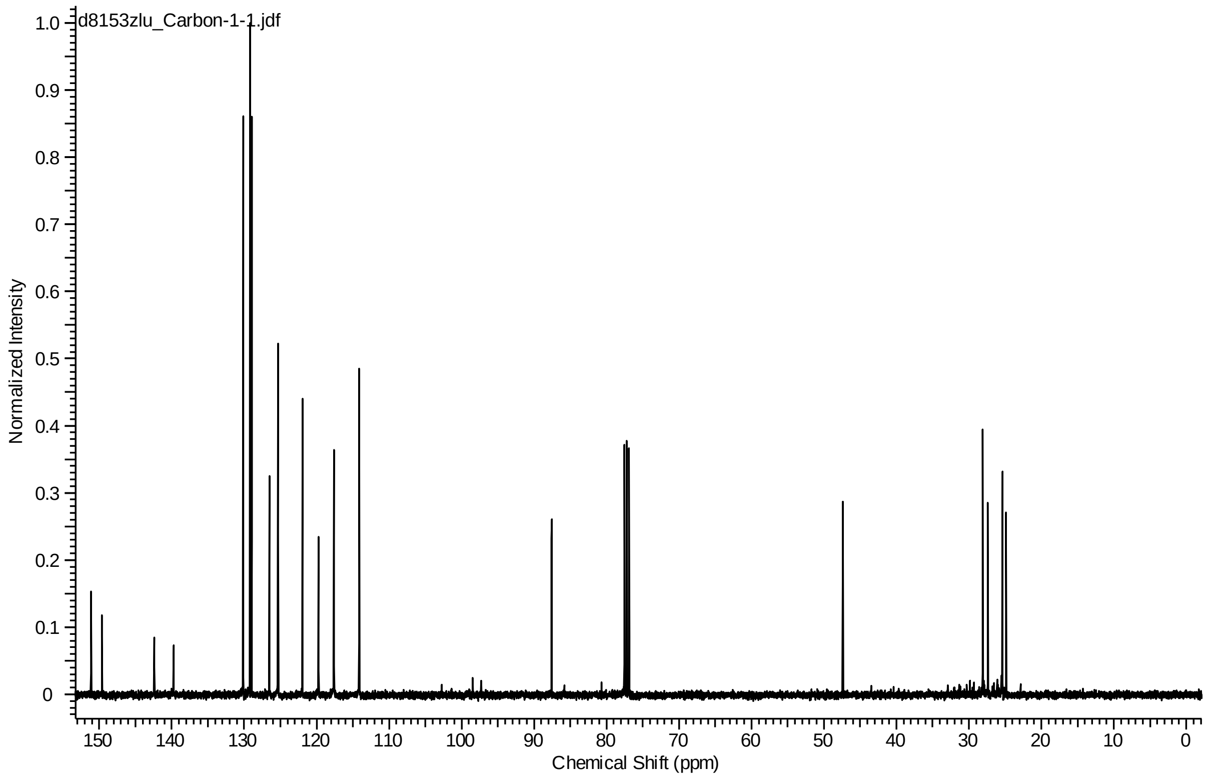

Supplementary Figure 43. ^{13}C NMR spectrum (100MHz) of **SI-5** in $CDCl_3$.

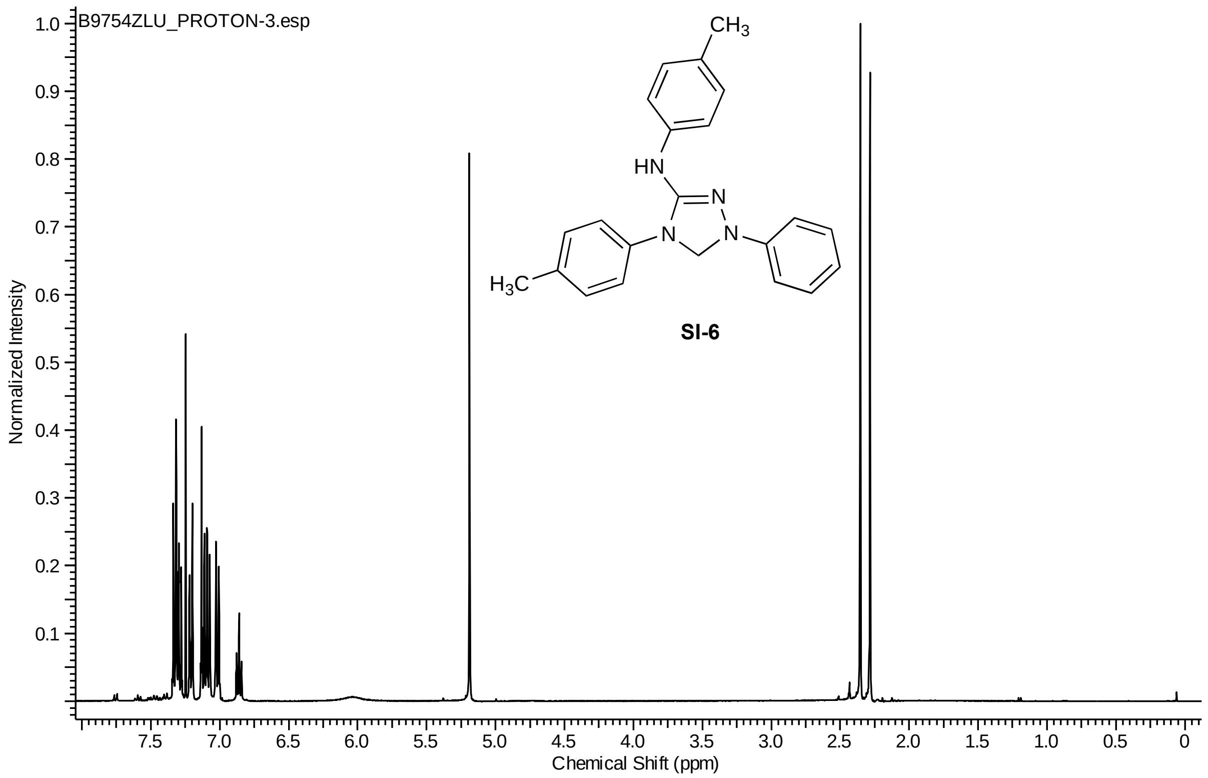

Supplementary Figure 44. ^{1}H NMR spectrum (400MHz) of **SI-6** in $CDCl_3$.

Supplementary Table 1. Comparison of selected bond lengths in Å of experimental and calculated geometries **3**, **4** and **8**. There are two independent molecules (**3a** and **3b**) in the crystal structure of **3**.

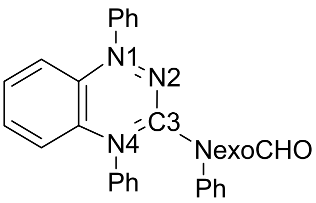

3

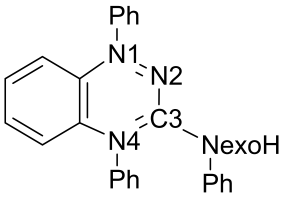

4

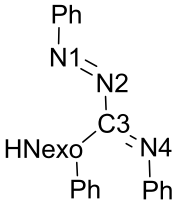

8

Bond	X-ray **3a**	X-ray **3b**	Calc **3**	X-ray **4**	Calc **4**	X-ray **8**	Calc **8**
N1-N2	1.3674(14)	1.3659(14)	1.364	1.3620(15)	1.367	1.2584(17)	1.251
N2-C3	1.3362(16)	1.3361(16)	1.336	1.3425(14)	1.335	1.4441(19)	1.439
C3-N4	1.3154(15)	1.3216(16)	1.322	1.3435(15)	1.338	1.2800(19)	1.285
C3-Nexo	1.4169(15)	1.4150(15)	1.414	1.3699(16)	1.379	1.3583(19)	1.366

Supplementary Table 2. Computed orbital contributions, spin densities and hyperfine coupling constants for N1, N2, N4 and *exo* nitrogen atoms in **1**, **3** and **4**.

	SOMO (%)			Spin (e)			hfcc (Gauss)			EPR-II (Gauss)		
Atom	**1**	**3**	**4**	**1**	**3**	**4**	**1**	**3**	**4**	**1**	**3**	**4**
N1	18	18	18	0.22	0.23	0.26	4.43	4.77	4.70	5.31	5.70	5.62
N2	20	21	19	0.37	0.46	0.33	3.27	3.47	3.28	4.11	4.32	4.16
N4	18	17	18	0.32	0.32	0.30	3.51	3.16	3.27	4.35	4.35	4.05
Nexo		1	0		0.01	-0.02		-0.17	-0.47		-0.20	-0.53

Supplementary Table 3. Orbital energies and orbital contributions in % for **1**, **3** and **4**. (Fused C = carbons present in both rings; C_4H_4 = carbons with attached hydrogens at C_6 ring of fused ring)

**1**		eV	N1	N2	C3	N4	Fused C	C_4H_4	Ph(N1)	Ph(C3)
Alpha SO										
78	α-L+2	-0.90	9	-3	7	0	2	0	85	0
77	α-L+1	-1.10	1	0	1	0	5	23	69	0
76	α-LUSO	-1.56	1	9	19	2	15	16	2	37
**75**	**α-HOSO**	**-4.81**	**18**	**20**	**0**	**18**	**13**	**21**	**8**	**1**
74	α-H-1	-6.57	3	2	2	7	3	26	3	54
73	α-H-2	-6.93	0	0	0	0	0	0	0	98
Beta SO										
78	β-L+3	-0.87	9	-3	7	0	2	0	84	1
77	β-L+2	-1.03	2	0	1	0	4	18	75	0
76	β-L+1	-1.43	1	13	14	1	17	17	2	34
75	β-LUSO	-2.79	16	20	1	21	6	25	9	2
74	β-HOSO	-6.32	4	1	6	8	3	29	4	44
73	β-H-1	-6.91	1	4	1	3	2	7	5	78

**3**		eV	N1	N2	C3	N4	Fused C	C_4H_4	Ph(N1)	Nexo	Ph(Nexo)	CHO
Alpha SO												
89	α-L+2	-1.01	6	0	7	0	2	3	81	0	1	1
88	α-L+1	-1.23	3	1	7	0	5	28	52	0	1	3
87	α-LUSO	-1.36	-1	7	13	1	24	25	22	1	1	9
**86**	**α-HOSO**	**-5.10**	**18**	**21**	**0**	**17**	**13**	**21**	**9**	**1**	**0**	**0**
85	α-H-1	-6.62	3	2	2	5	0	15	2	16	47	8
84	α-H-2	-7.01	0	0	1	0	0	0	0	0	88	10
Beta SO												
89	β-L+3	-0.97	5	0	7	0	3	6	75	0	1	2
88	β-L+2	-1.15	4	1	7	0	4	19	60	0	1	4
87	β-L+1	-1.28	0	9	9	0	23	25	25	0	0	8
86	β-LUSO	-3.06	17	23	0	18	5	24	9	2	0	1
85	β-HOSO	-6.42	4	1	5	8	2	24	3	17	28	8
84	β-H-1	-6.95	3	6	3	9	1	26	9	0	39	3

**4**		eV	N1	N2	C3	N4	Fused C	C_4H_4	Ph(N1)	Nexo	Ph(Nexo)	H(Nexo)
Alpha SO												
82	α-L+2	-0.84	2	2	9	0	12	13	49	0	13	0
81	α-L+1	-1.00	7	0	9	0	9	18	50	0	8	0
80	α-LUSO	-1.11	0	2	2	0	10	21	64	0	2	0
**79**	**α-HOSO**	**-4.81**	**18**	**19**	**1**	**18**	**15**	**21**	**8**	**0**	**0**	**0**
78	α-H-1	-5.77	2	5	1	5	1	9	1	23	53	0
77	α-H-2	-6.93	0	0	0	0	0	1	0	0	98	0
Beta SO												
82	β-L+3	-0.77	1	4	10	0	15	16	35	0	18	0
81	β-L+2	-0.94	8	0	6	0	6	11	64	0	5	0
80	β-L+1	-1.04	0	2	1	0	8	19	67	0	1	0
79	β-LUSO	-2.79	17	21	1	19	7	24	9	0	0	0
78	β-HOSO	-5.57	2	4	2	7	3	13	1	24	44	0
77	β-H-1	-6.76	5	4	4	4	2	49	13	1	18	0

Supplementary Table 4. Half-wave potentials for radicals **3** and **4**.

Radical	$E^{0/+1}_{½}$ (V)	$E^{-1/0}_{½}$ (V)	ΔE (V)
**3**	-0.11	-1.06	0.97
**4**	-0.32	-1.21	0.89

Supplementary Table 5. Peak table for UV-Visible spectrum of **3**.

λ_{max} (nm)	ε ($dm^3mol^{-1}cm^{-1}$)
560	9.25×10^2
533	9.53×10^2
471	9.34×10^2
370	5.00×10^3
320	7.00×10^3
254	3.42×10^4

Supplementary Table 6. Peak table for UV-Visible spectrum of **4**.

λ_{max} (nm)	ε ($dm^3mol^{-1}cm^{-1}$)
594	1.18×10^3
413	2.67×10^3
281	3.06×10^4

Supplementary Table 7. Crystal data and structure refinement parameters for **3**, **4** and **8**.

Compound	**3**	**4**	**8**
Empirical formula	$C_{20}H_{15}N_4O$	$C_{19}H_{15}N_4$	$C_{19}H_{16}N_4$

Formula weight	327.36	299.35	300.36
Temperature/K	120	120.0	120.0
Crystal system	triclinic	monoclinic	orthorhombic
Space group	P-1	$P2_1/c$	$P2_12_12_1$
a/Å	10.1989(4)	10.9766(9)	6.0615(2)
b/Å	10.5284(4)	5.1716(5)	8.3735(3)
c/Å	17.5186(7)	25.645(2)	31.2205(12)
α/°	96.7530(10)	90.00	90.00
β/°	101.3240(10)	100.205(3)	90.00
γ/°	117.5410(10)	90.00	90.00
Volume/$Å^3$	1587.80(11)	1432.8(2)	1584.62(10)
Z	4	4	4
$\rho_{calc}mg/mm^3$	1.369	1.388	1.259
m/mm^{-1}	0.088	0.086	0.077
F(000)	684.0	628.0	632.0
2Θ range for data collection	2.44 to 58°	4.5 to 56	5.22 to 56°
Reflections collected	26403	26184	24398
Independent reflections, R_{int}	8433, 0.0287	3445, 0.0745	3815, 0.0626
Data/restraints/parameters	8433/0/571	3445/0/178	3815/0/272
Goodness-of-fit on F^2	1.023	1.022	1.055
Final R_1 indexes [I>=2σ (I)]	0.0472	0.0637	0.0427
Final wR_2 indexes [all data]	0.1512	0.1684	0.0773

Largest diff. peak/hole / e Å^{-3}	0.37/-0.26	0.50/-0.52	0.17/-0.18
Flack parameter	-	-	0(2)

Supplementary Methods

General Instrumentation and Materials

NMR Spectra were obtained using Bruker Ultrashield 400 MHz, Varian Mercury 400 MHz and Varian VNMR 600 MHz NMR spectrometers operated at 25˚C. Liquid chromatography mass spectrometry (LC-MS) spectra were obtained using a Waters (UK) TQD mass spectrometer (low resolution ESI+, electrospray in positive ion mode, ES+).

Nitron was used as received from Sigma Aldrich (≥97% purity) and Tokyo Chemical Industries UK (≥98% purity) without further purification. Flash column chromatography was conducted using silica gel supplied from Sigma Aldrich and Fluorochem and monitored by thin layer chromatography (TLC) analysis. All synthetic solvents were obtained from Fisher Chemicals: methanol and acetonitrile were obtained as HPLC grade (99.9% min assay by GC); acetone and toluene were obtained as analytical reagent grade. $CDCl_3$ (99.8% D) was obtained from Apollo Scientific, and all other deuterated solvents (CD_3CN and CD_3OD (99.8% D)) were obtained from Cambridge Isotope Laboratories.

Computational Methods

All geometry optimisations of **1**, **3**, **4**, **6**, **8** and **9** were carried out at B3LYP[1], [2]/6-311+G(d,p) [3], [4] using the GAUSSIAN09[5] package and confirmed as true minima by frequency calculations. Comparison of observed geometries with X-ray geometries for **3**, **4** and **8** reveals very good agreements with differences of less than 0.01 Å (Supplementary Table 1).

Electronic structure (molecular orbital, spin density, EPR) data in the radical species **1**, **3** and **4** were obtained at the hybrid-DFT UB3LYP/6-311+G(d,p) with molecular orbitals and spin densities plotted using GABEDIT[6] (Supplementary Figures 3 and 8) and orbital contributions determined with GAUSSSUM (Supplementary Tables 2 and 3).[7] The SOMO in the radical species is assumed here to be the highest alpha(α) singly occupied molecular orbital, α-HOSO, where α- and β- single orbitals are treated as separate orbitals in open-shell calculations at UB3LYP/6-311+G(d,p). The model chemistry B3LYP/ EPR-II[8] was used on UB3LYP/6-311+G(d,p) optimised geometries as it is considered to be more suitable for predicting EPR parameters (Supplementary Table 2).

The rotational energy profiles for the triazabutadienes (**6**, **8** and **9**) were computed by constraining the N=N-C=N torsion angle at 10° intervals from 0° to 360° (Supplementary Fig. 10). These rotational energy profiles on **6**, **8** and **9** were computed to examine any preference for the *s-trans* conformer (N=N-C=N torsion angle at 180°) over the *s-cis* conformer (N=N-C=N at 0°). The *s-cis* conformer of **6**, **8** and **9** would potentially facilitate cyclisation to form **3**, **4** and **1** respectively. There is a preference for the *s-trans* in **8**, as found in the X-ray data for **8**, with a rotational energy barrier of 5.4 kcal mol^{-1}. This is due to a favorable hydrogen bond with a computed N…H-N distance of 2.06 Å. The more stable *s-trans* conformer in **8** could hinder the cyclisation to **4** from **8** assuming this is the barrier in the cyclisation pathway. A weaker preference is observed for the *s-trans* over the *s-cis* by 0.2 kcal mol^{-1} in **6**.

The triazabutadiene **9** has been explored computationally elsewhere[9] as the *s-cis* form (N=N-C=N at ca 70°) prior to cyclisation of **1** which clearly would aid cyclisation compared to the favoured *trans* conformer of **8**. However, the barrier to cyclisation to **1** from **9** was determined to be 38.6 kcal mol^{-1} which is much higher than the C-N bond rotation barrier of only 3.3 kcal mol^{-1} in the N=N-C=N unit of **9**. Given the similarities between **6**, **8** and **9**, the barrier to the formation of the cyclised **3** from **6** (and indeed **4** from **8**) is assumed here to be similarly high and the C-N bond rotation barrier does not play an important role in the cyclisation of these triazabutadienes.

Instrumental Methods

Electron Paramagnetic Resonance Spectroscopy.

EPR spectra were recorded on a Bruker EMX Micro spectrometer operating at X-band (9.5 GHz frequency), power 5 mW, modulation frequency 100 kHz, modulation amplitude 1 G. The spectra were recorded at room temperature using 0.1 mM solutions of radicals in degassed toluene.

Cyclic Voltammetry

A potentiostat (Ivium Compactstat.e) interfaced with a personal computer was used for the electrochemical measurements. A three-electrode cell containing a Pt wire pseudo-

reference electrode, a Pt foil (area = 1 cm^2) counter electrode and a Pt working electrode were used in a Faraday cage.

Cyclic voltammetry measurements were carried out on **3** and **4** as 1 mM quantities in Bu_4NBF_4 solutions (0.1 M) in degassed, dried acetonitrile (Supplementary Figs. 4 and 7). The electrolyte, Bu_4NBF_4, was dried before use and inert conditions were maintained during measurements. Decamethylferrocene (Cp^*_2Fe) and ferrocene (Cp_2Fe) were used as internal references for radicals **3** and **4** respectively. A scan rate of 50 mV/s was used for all measurements. All potentials (Supplementary Table 4) are quoted relative to the ferrocenium/ferrocene (Cp_2Fe^+/Cp_2Fe) couple. The decamethylferrocenium/decamethylferrocene ($Cp^*_2Fe^+/Cp^*_2Fe$) couple has a half-cell potential of -0.50 V relative to the Cp_2Fe^+/Cp_2Fe couple in this setup.

Infrared spectra

IR spectra were taken of solid samples of radicals **3**-**4** using a Perkin-Elmer Spectrum 2 infrared spectrophotometer (Supplementary Figures 13-14).

UV-visible spectra

UV-Visible spectra of radicals **3-4** were determined in acetonitrile solution using a Varian Cary 100 Bio UV-visible spectrophotometer at 25 °C (Supplementary Figure 15).

Single Crystal X-ray Crystallography

The X-ray single crystal data have been collected using λMoKα-radiation (λ=0.71073Å) at 120.0K for compound **3** on a Bruker SMART CCD 6000 (fine-focus sealed tube, graphite monochromator), for compound **4** on a Bruker D8Venture (Photon100 CMOS detector, IμS-microsource, focusing mirrors) and for compound **8** - on an Agilent Gemini S-Ultra (Sapphire-3 CCD detector, fine-focus sealed tube, graphite monochromator) diffractometers equipped with Cryostream (Oxford Cryosystems) open-flow nitrogen cryostat. The structures were solved by direct method and refined by full-matrix least squares on F^2 for all data using Olex2S[10] and SHELXTL[11] software. All non-hydrogen atoms were refined with anisotropic displacement parameters, H-atoms were located on the difference map and refined isotropically. The crystal data and details of the refinement are listed in Supplementary Table 7. Crystallographic data for the structures **3**, **4** and **8** have been

deposited with the Cambridge Crystallographic Data Centre as supplementary publication CCDC numbers 1411555, 1484332 and 1411554 respectively.

Synthetic Methods

Reaction of Nitron in a range of solvents:

Reaction of Nitron 2 in acetonitrile at room temperature

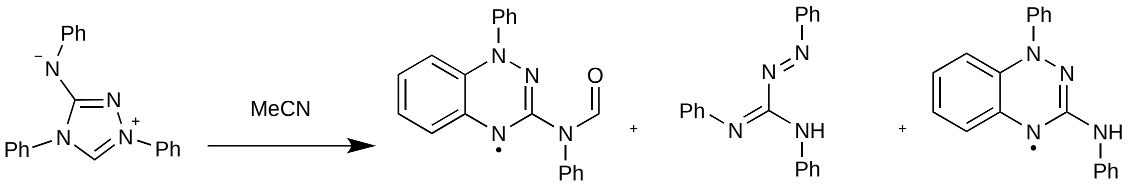

2 **3** **8** Trace **4** Trace

Nitron **2** (0.37 g, 1.2 mmol) was dissolved in acetonitrile (35 mL) and stirred with exposure to air for 24 hours at room temperature. The solution was then dried under reduced pressure to give crude black crystals.

The crystals were dissolved in minimum DCM and purified by column chromatography (98:2 DCM:MeOH). The major product was collected as a dark red fraction and dried under reduced pressure to yield amido radical **3** (0.23 g, 58%) as black crystals. m.p. 143-145 °C; $\mathbf{R}_{\boldsymbol{f}}$ = 0.62 (98:2 DCM:MeOH); **Elem. Anal.**: Calcd. For $C_{20}H_{15}N_4O$: C, 73.38; H, 4.62; N, 17.11; Found: C, 73.30; H, 4.63; N, 17.14; **UV-Vis** (nm, (ε, $dm^3mol^{-1}cm^{-1}$)): 254 (34,189), 320 (6998), 370 (5006), 471 (934), 533 (953), 560 (925); **IR**: 3070, 2925, 1681, 1586, 1483, 1370, 1204, 1077, 841, 760, 692, 612, 552, 495 cm^{-1}; **MS (ESI**, m/z, %): 327 (M^+, 100%), 328 ($M+H^+$, 69.4%), 329 ($M+2H^+$, 29.9%), 330 ($M+3H^+$, 4.0%).

Trace amounts (< 1%) of amino radical **4** and anilino-triazabutadiene **8** were also identified following column purification of crude product **3**.

Reaction of Nitron 2 in 50:50 v/v MeCN:H_2O at room temperature

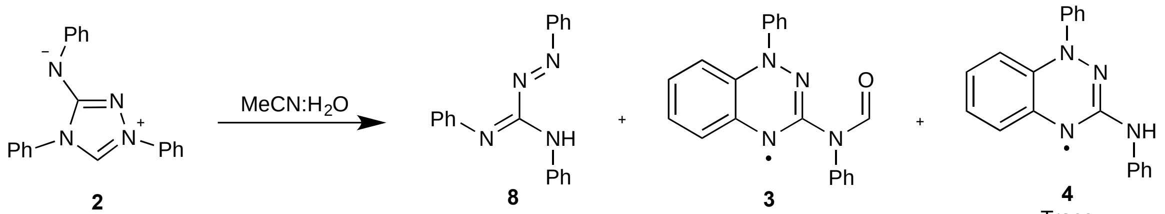

2 8 3 4

Nitron **2** (0.23 g, 0.68 mmol) was suspended in 50:50 v/v H_2O:MeCN (20 mL) and stirred with exposure to air for 24 hours at room temperature. The mixture changed overnight from a yellow suspension in an orange solution to a dark red solid suspended in a red solution. The mixture was concentrated and dried under reduced pressure to give a red solid, which was purified by column chromatography (98:2 DCM:MeOH). The major product isolated was the red triazabutadiene **8** (0.113 g, 51%). m.p. 107-108 °C; $\mathbf{R}_f$ = 0.35 (95:5 Hexane:EtOAc); **^{1}H NMR** (CDCl$_3$, 600 MHz,) δ = 7.95 (s, 1H, N-*H*), 7.86 (d, *J* = 8.0 Hz, 2H, Ar-*H*), 7.73-7.80 (m, 2H, Ar-*H*), 7.52-7.57 (m, 1H, Ar-*H*), 7.49 (t, *J* = 7.3 Hz, 2H, Ar-*H*), 7.37 (t, *J* = 7.7 Hz, 2H, Ar-*H*), 7.31 (t, *J* = 7.6 Hz, 2H, Ar-H), 7.02-7.15 (m, 4H, Ar-*H*); **^{13}C NMR** (CDCl$_3$, 600 MHz,) δ = 150.7, 148.8, 147.3, 138.7, 133.3, 129.4, 129.0, 128.0 124.4, 123.9, 123.1, 122.8, 119.1; **MS (ESI**, m/z): 301 (M+H).

Triazabutadiene **8** has been reported,[12], [13] however, has not been characterised to our knowledge.

Black amido radical **3** was isolated as minor product (0.064 g, 26%).

Trace amounts (< 1 mg) of dark green amino radical **4** were also collected.

Reaction of Nitron 2 in methanol at room temperature

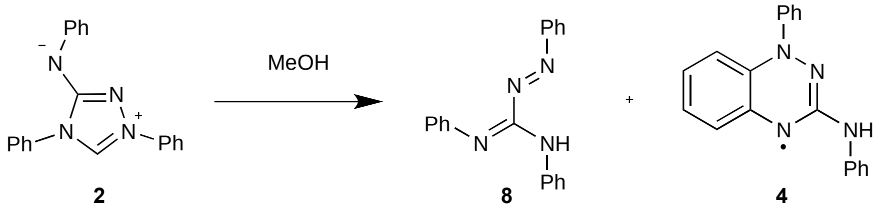

2 8 4

Nitron **2** (0.373 g, 1.19 mmol) was dissolved in methanol (35 mL) and stirred with exposure to air over 24 hours at room temperature. The solution changed colour during the reaction from yellow to dark red. The mixture was dried under reduced pressure to give a red solid.

The red solid was dissolved in a minimum amount of DCM and purified by column chromatography (98:2 DCM:MeOH). The major product was red triazabutadiene **8** (0.056 g, 15%).

Green amino radical **4** was also isolated (0.0074 g, 0.4%). **UV-Vis** (nm, (ε, $dm^3mol^{-1}cm^{-1}$)): 281 (30588), 413 (2675), 594 (1176) IR (cm^{-1}): 3305, 1598, 1530, 1481, 1419, 1339, 1214, 746, 690, 614, 486 cm^{-1}; **MS (ESI**, m/z): 299 [M^+]; **HRMS (ESI):** Calcd. For $C_{19}H_{15}N_4$: 299.1297; Found: 299.1298.

Reaction of Nitron 2 in toluene at room temperature

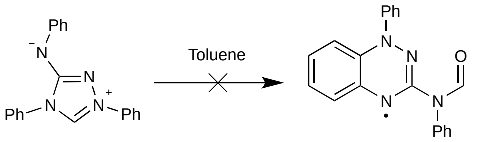

2 **3**

Nitron **2** (0.371 g, 1.18 mmol) was suspended in toluene (35 mL) and stirred with exposure to air over 24 hours at room temperature. The colour of the reaction mixture did not change over 24 hours. The toluene was evaporated under reduced pressure to yield a yellow powder of Nitron **2**. There was no evidence of any product formation by TLC.

Reaction of Nitron 2 in acetone at room temperature

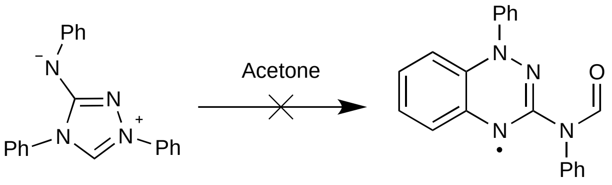

2 **3**

Nitron **2** (0.370 g, 1.19 mmol) was dissolved in acetone (35 mL) and stirred with exposure to air for 24 hours at room temperature. The reaction proceeded very slowly as observed by a slight change in colour, showing traces of radical **3** by TLC, however, no product was isolated.

Reaction of Nitron 2 in aqueous KOH solution in degassed acetonitrile

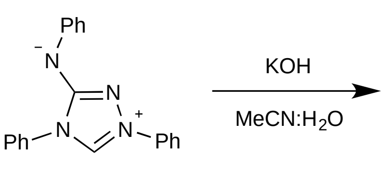

2 8

Nitron **2** (0.10 g, 0.32 mmol) was dissolved in degassed acetonitrile (10 mL) and added to a KOH solution (0.5 M, 20 mL). Immediately, a red oil formed above the aqueous layer. The solution was stirred for 48 hours and red crystals were precipitated. The product was collected by filtration and was purified by recrystallization, first from DCM and then from acetonitrile, to yield triazabutadiene **8** as red crystals (0.072 g, 75%).

The structure was confirmed by X-ray analysis.

Reaction of triazabutadiene 8 in acetonitrile at room temperature:

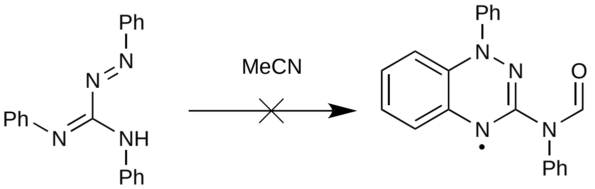

8 3

Triazabutadiene **8** (0.030 g, 0.099 mmol) was dissolved in acetonitrile (10 mL) and left exposed to air for one week. The colour of the solution did not change from light red over this period. No change was recorded by mass spectrometry or by TLC.

Preparation of amino radical 4:

Preparation of amino radical 4 by hydroxide-promoted hydrolysis

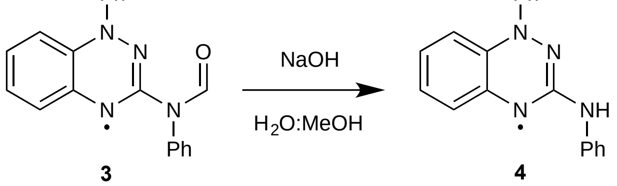

3 4

Radical **3** (0.39 g, 1.19 mmol) was dissolved in a 1 M NaOH solution in 50:50 H_2O:MeOH (40 mL) and stirred for 72 hours. The solvent was removed under reduced pressure and the

green residue dissolved in DCM (100 mL), washed with 1M HCl (1 x 50 mL), sat. $NaHCO_3$(aq) (1 x 50 mL), water (3 x 50 mL) and brine (1 x 50 mL). The organic layer was dried over Na_2SO_4, filtered and dried *in vacuo*. The residue was dissolved in the minimum volume of DCM and purified by column chromatography (98:2 DCM:MeOH). The product containing fractions were collected and dried *in vacuo* to give radical **4** (0.25 g, 70 %). The structure was confirmed by independent X-Ray analysis. m.p. 151-154 °C; $\mathbf{R_f}$ = 0.45 (98:2 DCM:MeOH); **MS (ESI**, m/z, %): 299 (M^+, 100), 300 ($M+H^+$, 72.49), 301 ($M+2H^+$, 12.7); **HRMS (ESI):** Calcd. For $C_{19}H_{15}N_4$: 299.1297; Found: 299.1294.

Preparation of amino radical 4 by acid-catalysed hydrolysis

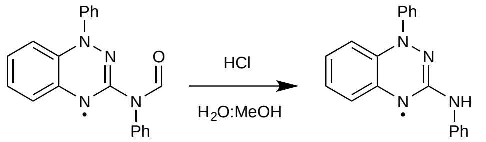

3 4

Radical **3** (0.250 g, 1.19 mmol) was dissolved in a 5 M HCl solution in 50:50 H_2O:MeOH (40 mL) and stirred for 2 hours. The solvent was removed under reduced pressure and the green residue dissolved in CH_2Cl_2 (100 mL) and washed with 1 M Na_2CO_3 (1 x 50 mL) then water (3 x 50 mL) and finally brine (1 x 50 mL). The organic layer was dried over Na_2SO_4, filtered and dried *in vacuo*. The residue was dissolved in the minimum volume of CH_2Cl_2 and purified by column chromatography (98:2 CH_2Cl_2:MeOH) The product containing fractions were collected and dried *in vacuo* to give radical **4** (0.098 g, 43%). The structure was confirmed by independent X-ray analysis.

Synthesis of Nitron derivatives 11, 12, 13a, 13b:

Preparation of 2,4-diphenyl-5-phenylamino-1,2,4-triazole derivatives

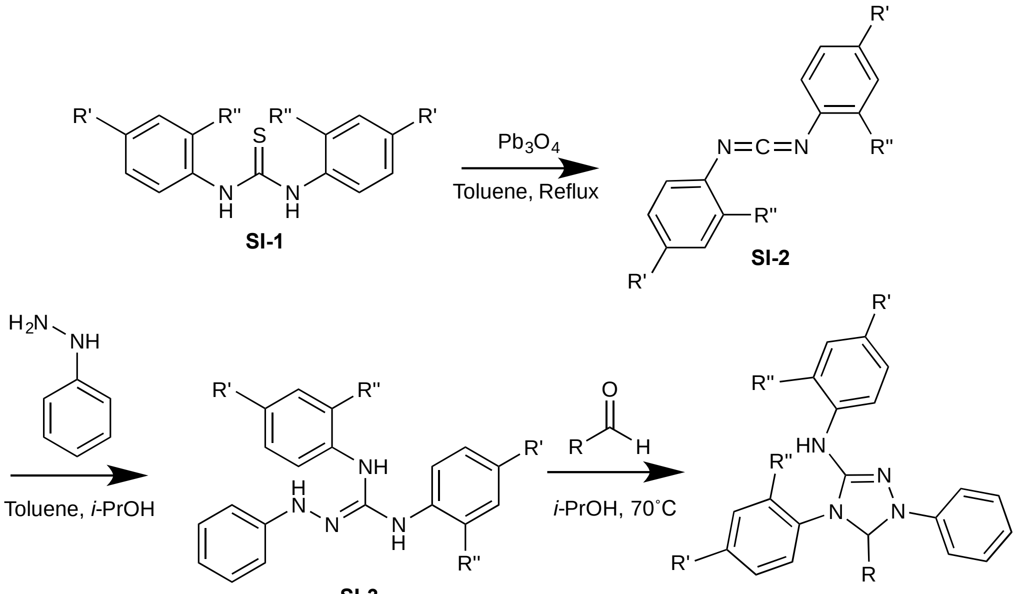

SI-4 R = Pr, R'= R'' = H
SI-5 R = Cyclopentyl R'= R'' = H
SI-6 R = H, R' = CH_3, R'' = H
SI-7 R = H, R' = H, R'' = CH_3

The preparation of 2,4-diphenyl-5-phenylamino-1,2,4-triazole derivatives **SI-4, SI-5, SI-6** and **SI-7** were carried out following the modification of a literature procedure [14] described below for the synthesis of 2,4-diphenyl-5-phenylamino-3-propyl-1,2,4-triazole **SI-4**.

Lead oxide (Pb_3O_4, 5 g, 7 mmol) was added to a solution of diphenylthiourea **SI-1** (R'=R''=H) (5 g, 22 mmol) in toluene (50 mL). The mixture was refluxed until water ceased to liberate (Dean-Stark trap). The mixture was then filtered and solvent was removed under reduced pressure. The diphenyl carbodiimide residue was used for the next step without purification.

Phenylhydrazine (2.3 mL, 2.1 g, 19 mmol) was added dropwise to a solution of the N,N-diphenylcarbodiimide residue **SI-2** (R'=R''=H) in toluene (10 mL) at 0 °C. Isopropanol (30 mL) was then added to the mixture, and the resulting mixture was refrigerated overnight. The crystalline product was then filtered off and dried in air. The crude product was used directly for next step without further purification.

To a suspension of triphenylaminoguanidine **SI-3** (R'=R''=H) (2.0 g, 6.6 mmol) in isopropanol (10 mL), butanal (0.94 g, 13 mmol) was added. The mixture was then heated to 70 °C for 2 h and cooled to room temperature. The solid was filtered off, washed with isopropanol (10 mL), and dried in air. The product 2,4-diphenyl-5-phenylamino-3-propyl-1,2,4-triazole **SI-4** was used directly for the next step without further purification (1.80 g, 23%).

<u>**SI-4** 2,4-Diphenyl-5-phenylamino-3-propyl-1,2,4-triazole</u>

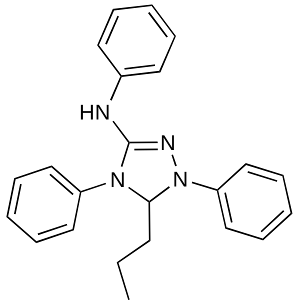

SI-4

^{1}**H NMR** ($CDCl_3$, 400 MHz) δ = 7.44 (d, *J* = 8.7 Hz, 2H, Ar-*H*), 7.32 (t, *J* = 8.2 Hz, 2H, Ar-*H*), 7.17-7.28 (m, 5H, Ar-*H*), 7.13 (d, *J* = 8.7 Hz, 2H, Ar-*H*), 7.04 (d, *J* = 7.8 Hz, 2H, Ar-*H*), 6.92 (t, *J* = 7.3 Hz, 1H, Ar-*H*), 6.79 (t, *J* = 7.3 Hz, 1H, Ar-*H*), 5.25 (t, *J* = 4.6 Hz, 1H, NC*H*N), 1.81-1.89 (m, 2H, CH_2), 1.50-1.69 (m, 2H, CH_2), 0.91 (t, *J* = 7.3 Hz, 3H, CH_3); ^{13}**C NMR** ($CDCl_3$, 400 MHz) δ = 149.6, 147.9, 140.7, 139.7, 130.2, 129.2, 129.1, 126.7, 125.4, 121.9, 119.4, 117.6, 113.8, 84.4, 37.7, 16.8, 14.2; **MS** (**ESI**, m/z, %): 357.2046 (M+H$^+$, 100).

SI-5 2,4-Diphenyl-5-phenylamino-3-cyclopentyl-1,2,4-triazole

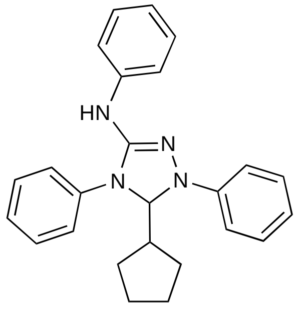

SI-5

Using the general procedure above and cyclopentanecarboxaldehyde (1.27 g, 13 mmol), yielded the product 2,4-diphenyl-5-phenylamino-3-cyclopentyl-1,2,4-triazole **SI-5** (1.09 g, 18%). The product was used directly in the next step without further purification.

^{1}H NMR ($CDCl_3$, 400 MHz) δ = 7.51-7.6 (m, 10H, Ar-*H*), 7.38-7.36 (m, 2H, Ar-*H*), 7.11-7.15 (m, 2H, Ar-*H*), 6.71 (tt, *J* = 7, 1 Hz, 1H, Ar-*H*), 5.27 (d, *J* = 5 Hz, 1H, NC*H*N), 2.98-3.07 (m, 1H, NCHC*H*), 1.80-1.86 (m, 2H, CHCH_2), 1.59-1.70 (m, 2H, CHCH_2), 1.19-1.41 (m, 4H, 2 CH_2CH_2); **^{13}C NMR** ($CDCl_3$, 400 MHz) δ = 150.8, 149.3, 142.1, 139.4, 129.7, 128.8, 128.6, 126.2, 125.0, 121.6, 119.4, 117.2, 113.7, 87.2, 47.0, 27.7, 27.0, 25.0, 24.5; **MS** (**ESI**, m/z, %): 383.2207 ($M+H^+$, 100%).

SI-6 2-Phenyl-4-*p*-tolyl-5-*p*-tolylamino-1,2,4-triazole

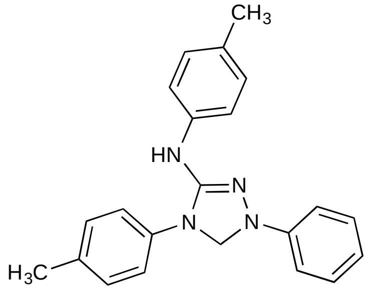

SI-6

Using the general procedure above with di-*p*-tolylthiourea (5 g, 22 mmol) and formaldehyde (37% soln. in H_2O, 2 mL, 20 mmol) yielded the product **SI-6** (1.55 g, 23%). The product was used directly in the next step without further purification.

SI-7 2-Phenyl-4-*o*-tolyl-5-*o*-tolylamino-1,2,4-triazole

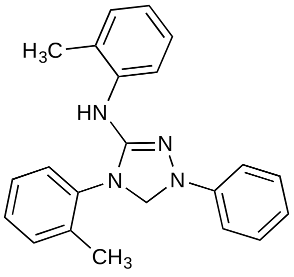

SI-7

Using the general procedure above using di-*o*-tolylthiourea (5 g, 22 mmol) and formaldehyde (37% soln. in H_2O, 2 mL, 20 mmol) yielded the product **SI-7** (1.63 g, 24 %). The product was used directly in the next step without further purification.

Preparation of 2,4-diphenyl-5-phenylamido-1,2,4-triazolium (Nitron) derivatives:

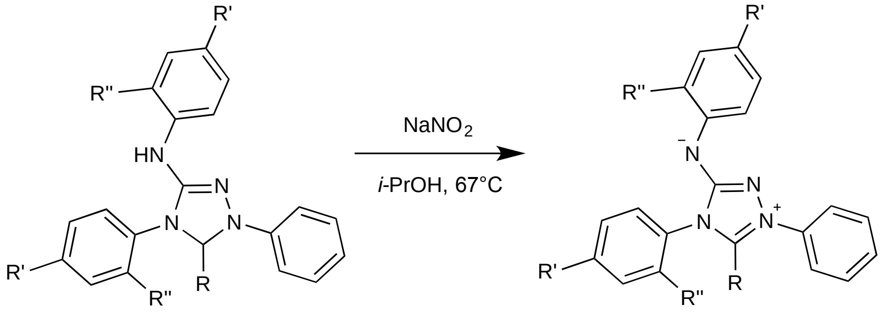

SI-4, SI-5, SI-6, SI-7

11 R = Pr, R' = R'' = H,
12 R = Cyclopentyl, R' = R'' = H,
13a R = H, R' = CH_3, R'' = H
13b R = H, R' = CH_3, R'' = H

The oxidation of triazoles to Nitron derivatives **11**, **12**, **13a** and **13b** was carried out following the modification of a literature procedure described below[14] for the synthesis of Nitron derivative **11**.

To a suspension of 2,4-diphenyl-5-phenylamino-3-propyl-1,2,4-triazole **SI-4** (130 mg, 0.37 mmol) in isopropanol (0.6 mL), glacial acetic acid (0.1 mL) was added at 67 °C. Then a solution of sodium nitrite (48.8 mg, 0.7 mmol) in H_2O (110 µL) was added. The mixture was stirred for 10 min, and then 35% ammonia aqueous solution was added at 0 °C. The precipitate was collected by filtration, dried in a vacuum oven to yield Nitron derivative **11** (0.09 g, 70%).

11 2,4-Diphenyl-5-anilino-3-propyl-1,2,4-triazolium

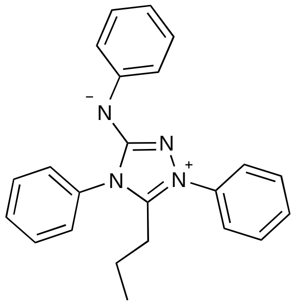

11

m.p. 220-221 °C; **^{1}H NMR** ($CDCl_3$, 400 MHz) δ = 7.67-7.69 (m, 2H, Ar-*H*), 7.42-7.60 (m, 10H, Ar-*H*), 7.14-7.18 (m, 2H, Ar-*H*), 6.73 (tt, *J* = 7, 1 Hz, 1H, Ar-*H*), 2.73 (t, *J* = 8 Hz, 2H, NCCH_2), 1.25-1.34 (m, 2H, CH_2CH_2), 0.70 (t, *J* = 8 Hz, 3H, CH_3); **^{13}C NMR** ($CDCl_3$, 400 MHz) δ = 155.9, 150.1, 145.4, 136.6, 133.3, 130.1, 129.8, 129.6, 128.4, 128.3, 125.7, 122.3, 118.3, 26.0, 20.0, 13.6; **MS** (**ESI**, m/z, %): 355.1905 (M+H$^+$, 100%).

12 2,4-Diphenyl-5-anilino-3-cyclopentyl-1,2,4-triazolium

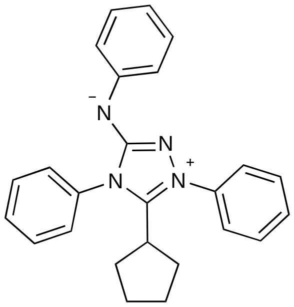

12

As with the general procedure above, 2,4-diphenyl-5-anilino-3-cyclopentyl-1,2,4-triazole **SI-5** (130 mg, 0.23 mmol) was oxidised using sodium nitrite (48.8 mg, 0.7 mmol), in H_2O and isopropanol yielding Nitron derivative **12** (87.9 mg, 68%). m.p. 244-245 °C; **^{1}H NMR** ($CDCl_3$, 400 MHz) δ = 7.52-7.62 (m, 10H, Ar-*H*), 7.38-7.40 (m, 2H, Ar-*H*), 7.11-7.15 (m, 2H, Ar-*H*), 6.71 (tt, *J* = 7.1 Hz, 1H, Ar-*H*), 2.97-3.06 (m, 1H, C*H*), 1.78-1.86 (m, 2H, CHCH_2), 1.59-1.69 (m, 2H, CHCH_2), 1.20-1.40 (m, 4H, $2CH_2CH_2$); **^{13}C NMR** ($CDCl_3$, 400 MHz) δ = 155.6, 149.0, 147.6,

136.7, 132.8, 130.7, 129.9, 129.8, 129.4, 128.4, 126.7, 121.7, 119.4, 35.6, 31.1, 25.9; **MS** (**ESI**, m/z, %): 381.2026 (M+H$^+$, 100).

<u>**13a** 2-Phenyl-4-*p*-tolyl-5-*p*-methylanilino-1,2,4-triazolium</u>

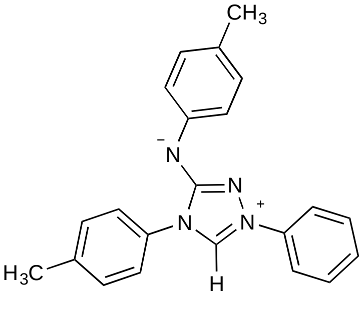

13a

2-Phenyl-4-*p*-tolyl-5-*p*-tolylamino-1,2,4-triazole **SI-6** (128 mg, 0.37 mmol) was oxidised using sodium nitrite (48.8 mg, 0.7 mmol) in H_2O and isopropanol yielding Nitron derivative **13a** (81.4 mg, 63%). m.p. 151-153 °C; **^{1}H NMR** ($CDCl_3$, 400 MHz) δ = 6.99-7.86 (m, 14H, Ar-*H* and C*H*), 2.25 (s, 3H, CH_3), 2.22 (s, 3H, CH_3); **MS** (**ESI**, m/z, %): 341.1743 (M+H+, 100).

The sample was unstable and further characterization was not attempted

<u>**13b** 2-Phenyl-4-o-tolyl-5-o-methylanilino-1,2,4-triazolium</u>

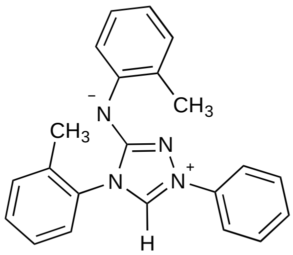

13b

2-Phenyl-4-*o*-tolyl-5-*o*-tolylamino-1,2,4-triazole **SI-7** (100 mg, 0.29 mmol) was oxidised using sodium nitrite (41.4 mg, 0.6 mmol) in H_2O and isopropanol yielding Nitron derivative **13b** (78.5 mg, 79%). m.p. 165-166 °C; **^{1}H NMR** ($CDCl_3$, 400 MHz) δ = 6.72-7.84 (m, 14H, Ar-*H* and C*H*), 2.39 (s, 3H, CH_3), 2.11 (s, 3H, CH_3); **MS** (**ESI**, m/z, %): 341.1766 (M+H+, 100%).

The NMR lines were broadened due to the presence of Blatter-type radical formed spontaneously from **13b**. The sample was unstable and further characterization was not attempted.

Preparation of Blatter-type radicals from Nitron derivatives.

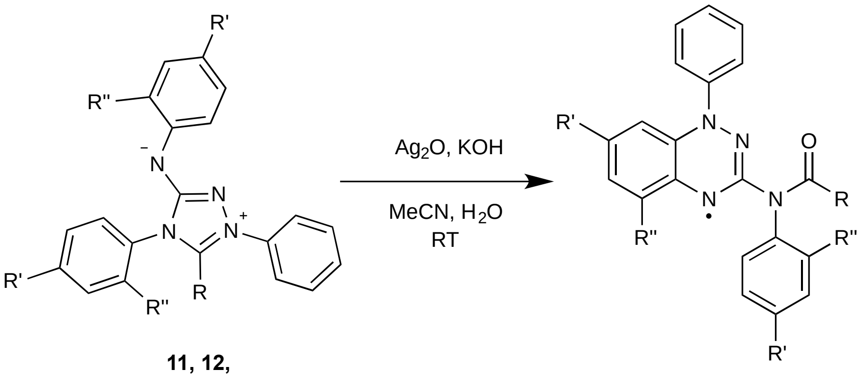

11, 12,
13a, 13b

14 R = Pr, R' = R'' = H
15 R = Cyclopentyl, R' = R'' = H
16a R = H R' = CH$_3$, R'' = H
16b R = H, R' = H, R'' = CH$_3$

Blatter-type radicals (**14, 15, 16a** and **16b**) were prepared using the representative procedure described below for the synthesis of **14**.

To a solution of Nitron derivative **11** (106 mg, 0.3 mmol) in acetonitrile (20 mL), silver oxide (75 mg, 0.3 mmol) and aqueous KOH solution (0.5 M, 100 µL) were added. The mixture was stirred at room temperature overnight. The mixture was then filtered and solvent was removed under reduced pressure and the residue was purified by column chromatography ($CHCl_3$: MeOH = 30 : 1, R*f* = 0.3) yielding the product **14** (43.8 mg, 42%).

Radical **14**

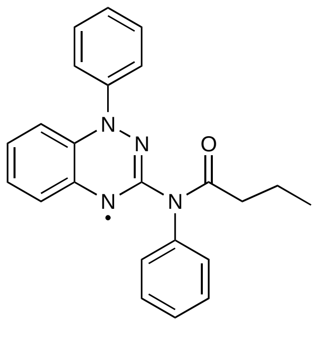

14

m.p.: decomposes above 150 °C; **Elemental Analysis**: Calcd. For $C_{23}H_{22}N_4O$: C, 74.77; H, 5.73; N, 15.17; Found: C, 74.53; H, 5.90; N, 14.95; **HRMS (ESI):** Calcd. For $C_{23}H_{22}N_4O$: 370.1788; Found: 370.1791.

Preparation of **15**

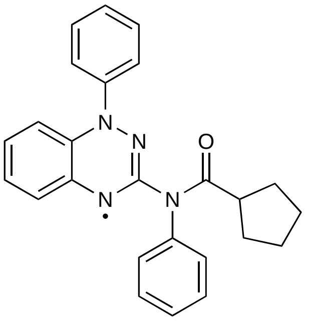

15

Following the general procedure above, Nitron derivative **12** was reacted to form the radical **15** (47.8 mg, 46%). m.p.: decomposes above 150 °C; **Elemental Analysis**: Calcd. For $C_{25}H_{23}N_4O$: C, 75.93; H, 5.86; N, 14.17; Found: C, 75.77; H, 5.45; N, 13.89; **HRMS (ESI):** Calcd. For $C_{25}H_{23}N_4O$: 396.1945; Found: 396.1947; Calcd. For $C_{25}H_{23}N_4ONa$: 418.1764, Found: 418.1763.

Preparation of **16a**

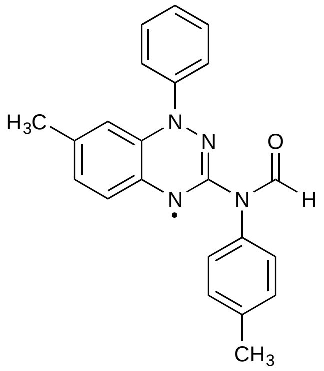

16a

Following the general procedure above, Nitron derivative **13a** (100 mg, 0.31 mmol) was reacted to form the radical **16a** (69.9 mg, 67%). m.p.: decomposes above 150 °C; **Elemental Analysis**: Calcd. For $C_{22}H_{19}N_4O$: C, 74.35; H, 5.39; N, 15.76; Found: C, 73.57; H, 5.41; N, 15.70; **HRMS (ESI):** Calcd. For $C_{22}H_{20}N_4O$: 356.1632; Found: 356.1636; Calcd. For $C_{22}H_{19}N_4ONa$: 378.1451; Found: 378.1456.

Preparation of **16b**

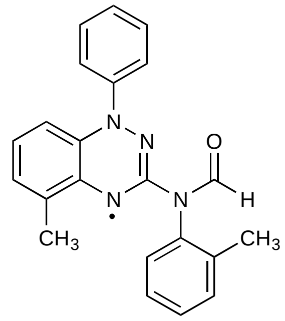

16b

Following the general procedure above, Nitron derivative **13b** (100 mg, 0.31 mmol) was reacted to form the radical **16b** (64.5 mg, 62%). m.p.: decomposes above 150 °C; **Elemental Analysis**: Calcd. For $C_{22}H_{19}N_4O$: C, 74.35; H, 5.39; N, 15.76; Found: C, 74.47; H, 5.41; N, 15.69; **HRMS (ESI):** Calcd. For $C_{22}H_{20}N_4O$: 356.1632; Found: 356.1633; Calcd. For $C_{22}H_{19}N_4ONa$: 378.1451; Found: 378.1457.

Supplementary References

[1] Becke, A.D. Density-functional thermochemistry. 3. The role of exact exchange. *J. Chem. Phys.* **98**, 5648–5652 (1993).

[2] Lee, C., Yang, W. & Parr, R. G. Development of the Colle-Salvetti correlation-energy formula into a functional of the electron density. *Phys. Rev. B* **37**, 785–789 (1988).

[3] Petersson, G. A. & Al-Laham, M. A. A complete basis set model chemistry. 2. Open-shell systems and the total energies of the 1st-row atoms. *J. Chem. Phys.* **94**, 6081–6090 (1991).

[4] Petersson, G. A., Bennett, A., Tensfeldt, T. G., Al-Laham, M. A., Shirley, W. A. & Mantzaris, J. A complete basis set model chemistry. 1. Open-shell systems and the total energies of the 1st-row atoms and hydrides of the 1st row elements. *J. Chem. Phys.* **89**, 2193–2218 (1988).

[5] GAUSSIAN09, Revision A.02, M. J. Frisch et al, *Gaussian, Inc.*, Wallingford CT, 2009.

[6] Allouche, A. R. Gabedit-A graphical user interface for computational chemistry softwares. *J. Comput. Chem.* **32**, 174–182 (2011).

[7] O'Boyle, N. M., Tenderholt, A. L. & Langner, K. M. cclib: A library for package-independent computational chemistry algorithms. *J. Comput. Chem.* **29**, 839–845 (2008).

[8] Barone, V. Recent advances in density functional methods, Part I, Ed. Chong, D. P., World Scientific Publ. Co., Singapore (1996).

[9] Bodzioch, A., Zheng, M., Kaszynski P. & Utecht, G. Functional group transformations in derivatives of 1,4-dihydrobenzo[1,2,4]triazinyl radical. *J. Org. Chem.* **79**, 7294-7310 (2014).

[10] Dolomanov, O. V., Bourhis, L. J., Gildea, R. J., Howard, J. A. K. & Puschmann, H. *J.* OLEX2: a complete structure solution, refinement and analysis program. *Appl. Cryst.* **42**, 339 (2009).

[11] Sheldrick, G. M. A short history of SHELX. *Acta Cryst.* **A64**, 112 (2008).

[12] Marckwald, W. & Wolff, P. Ueber das diphenylanilguanidin. *Chem. Ber.* **25**, 3116-3119 (1892).

[13] Raiford, L. C. & Daddow, W. T. The reactions of some carbonyl compounds with phenylhydrazine. *J. Am. Chem. Soc.* **53**, 1552 (1931).

[14] Kriven'ko, A. & Morozova, N. Synthesis of 1,4-diphenyl-3-phenylimino-1,2-dihydro-1,2,4-triazolium hydroxide (Nitron). *Russ. J. Appl. Chem.* **79**, 506-507 (2006).
